# Being facially expressive is socially advantageous

**DOI:** 10.1038/s41598-024-62902-6

**Published:** 2024-06-13

**Authors:** Eithne Kavanagh, Jamie Whitehouse, Bridget M. Waller

**Affiliations:** Department of Psychology, Nottingham Trent University, Nottingham, UK

**Keywords:** Psychology, Human behaviour, Evolution, Social evolution

## Abstract

Individuals vary in how they move their faces in everyday social interactions. In a first large-scale study, we measured variation in dynamic facial behaviour during social interaction and examined dyadic outcomes and impression formation. In Study 1, we recorded semi-structured video calls with 52 participants interacting with a confederate across various everyday contexts. Video clips were rated by 176 independent participants. In Study 2, we examined video calls of 1315 participants engaging in unstructured video-call interactions. Facial expressivity indices were extracted using automated Facial Action Coding Scheme analysis and measures of personality and partner impressions were obtained by self-report. Facial expressivity varied considerably across participants, but little across contexts, social partners or time. In Study 1, more facially expressive participants were more well-liked, agreeable, and successful at negotiating (if also more agreeable). Participants who were more facially competent, readable, and perceived as readable were also more well-liked. In Study 2, we replicated the findings that facial expressivity was associated with agreeableness and liking by their social partner, and additionally found it to be associated with extraversion and neuroticism. Findings suggest that facial behaviour is a stable individual difference that proffers social advantages, pointing towards an affiliative, adaptive function.

## Introduction

“Face-to-face” is the primary way humans have interacted with each other for potentially millions of years. In our everyday social interactions, our faces, and those of others, are moving. Facial movement is commonly assumed to perform a primarily emotional communication function, but there is a wealth of information that can be exchanged via facial movement, and we use this exchange to navigate varying social landscapes. Atypical facial expression production can be associated with difficulties in social functioning (e.g., autism^1^, Parkinson’s Disease^2^; facial paralysis^3^). It is, therefore, possible that facial behaviour may have a significant impact on the outcomes of our social lives. Humans have the most complex facial musculature system of any animal and even show unique variation in the presence of some facial muscles^4^. While cultural similarities and differences in facial expressions have been examined empirically e.g.,^5,6^, human population level individual differences in actual facial behaviour have not. Recent technological advances in automated facial coding have facilitated the quantification of facial behaviour in typical social interactions, a task that was previously too time-consuming to be feasible in a large sample. It is now possible, therefore, to take what should arguably be one of the first steps towards understanding any behaviour: quantitative observation.

While various aspects of facial perception have been heavily studied over decades e.g.,^7–9^, the study of facial production is essentially in its infancy. Twenty-three years ago, Preuschoft stated that “ethological work on the spontaneous use of facial expressions and their functions in the context of natural social interactions is still badly needed”^10, p. 261]^. We argue that this is still the case today. Our understanding of facial behaviour and its function is based almost exclusively on self-report questionnaires e.g.,^11^ or laboratory studies in which participants’ faces are observed, but in narrow windows outside of the social interaction in which they are thought to function. Some prevalent methods involve participants posing specific facial expressions e.g.,^12,13^, watching videos alone while their facial reactions are recorded e.g.,^14,15^ or performing tasks which do not involve interacting with a social partner^16,17^. The highly controlled nature of such studies makes them valuable contributions in understanding the mechanisms of facial behaviour. However, an over-reliance on these approaches creates gaps and biases in our understanding of its function, its costs and its benefits. These can only be addressed by examining how people behave in-situ and the direct impact of this behaviour.

Progress on understanding the social role of faces has also been hindered by an over-focus on the emotional content of facial behaviour^18^. The traditional approach to understanding facial communication views facial expressions as signals of internal emotional state and emphasises the similarity of facial configurations of emotions across humans^19,20^. Under this ‘Basic Emotions Theory’, expressivity refers to the tendency to express emotions outwardly, such as smiling to express happiness^19^. However, there is increasing understanding of the diversity of how such configurations are understood by others^9^, and that facial movements are not limited to expressing emotion alone, or indeed inextricably linked to emotions at all^21^. The conclusion that facial behaviour functions simply to express emotion does not address why this would have evolved and what social benefits these expressions may have. Humphrey^22^ argued that behavioural expression is a social act which can only be understood within a social context, and thus it is essential to incorporate its effect on the receiver when studying its function. It is the receiver who has potential to provide social benefits to the producer. Alternative approaches such as the Behavioural Ecological View^23^ consider facial expressions to be social tools that communicate motivations, intentions, or potential actions that are not necessarily tied to emotions. This view shifts our perspective of what facial expressivity means and how we should approach understanding individual differences in this behaviour. This does not *exclude* the possibility that facial movements may convey emotion, but it nevertheless does not make this assumption. Viewing facial production as a means to achieving social goals suggests that instead of measuring emotion, we should be examining social interaction consequences to understand meaning, such as the affiliative and competitive outcomes of social encounters. Doing so will provide insight into the factors that drive the evolution of facial behaviour and its variation in humans.

Historically, the study of facial behaviour has been heavily biased towards focusing on universality rather than variation. The literature is dominated by a focus on specific facial configurations that are considered to be universal across populations^6,20^. This has led to variation in facial behaviour being almost entirely ignored, and it is currently an open question whether facial expressivity can be considered an individual trait. The meaning of ‘trait’ and the criteria used to demonstrate ‘trait-likeness’ is debated and inconsistent across fields^24^. However, it is generally agreed that at least some degree of variation in a given behaviour, morphological feature or cognitive processing tendency should be explained by the individual, rather than entirely dependent on the environment or another level of organization, for it to be considered a trait^25,26^. This is sometimes referred to as the ‘person-situation’ debate (i.e., whether variation in behaviour is determined more by the individual or the context), which Fleeson^27^ declared to be at an end within personality research, arguing that both person and situation are contributing factors to variation in personality behaviour. However, this has not been fully addressed within facial behaviour research. Cohn et al.,^14^ found that individuals used some facial movements a similar amount in clinical interviews at two different time points. Ilgen et al.^16^ also conducted structured interviews and identified patterns of facial muscle movement (e.g., smiling with narrowed eyelids and raised cheeks) that people tended to use differentially. Specifically, use of particular combinations predicted self-reported social and emotional styles, such as being more compromising. These two studies point towards the potential for aspects of facial behaviour being stable individual differences. We don’t yet know, however, the extent of variation in facial expressivity (i.e., more frequent or diverse use of facial movements) in free-flowing, naturalistic interactions which are more reflective of every-day encounters such as meeting a stranger. Whether or not facial expressivity can be considered a stable individual difference can be determined by empirically measuring the level of consistency in individuals’ behaviour across typical social contexts, with different social partners and across time. By examining this stability, in tandem with examining the social consequences of facial behaviour, we can begin to understand its function in a real-world context.

### Facial expressivity in affiliative contexts

Many of our social interactions include a motivation to affiliate with a social partner, and facial expressivity could facilitate this social goal. There is likely to be strong selective pressure on traits that improve affiliation with others, as the development of a strong social network is predictive of higher survival and reproductive success, decreased mortality risk, and a broad range of positive health indicators^28–31^. Facial expression may promote affiliation and lead to greater social success. Based on short video-recordings (without a social partner), people who were more emotionally expressive (i.e., tend to behaviourally communicate internal emotions outwardly such as by laughing or crying^32^) and more generally facially expressive during a non-social experimental task^17^ were more well-liked by third-party observers. Fultz et al.,^33^ found that self- and other-reported nonverbal expressivity was related to greater liking in new and more established relationships. Riggio^34^ also found that more emotionally expressive people had more close friends and daily acquaintances than less expressive people. Such studies suggest there are positive social outcomes of facial expressivity.

Under the Behavioural Ecology View of Facial Expression^23^, whereby facial expressions can be understood as communicating intentions, a preference for more expressive social partners could be explained by facial expressions providing enhanced predictability. The social value of facial expressivity could be in allowing others to predict one’s behaviour^35^. More predictable social partners could be preferred by others by providing opportunity to respond optimally to their intentions, whether the intentions are advantageous or disadvantageous to the receiver, and facial expressivity could facilitate predicting one’s behaviour in real time. More expressive people are more judgeable (i.e., others can more accurately assess their personality^36^), and judgeability predicted greater liking in first impressions and long-term^37,38^ as well as relationship satisfaction in married couples^39^. Some research also indicates that individuals whose minds are easier to read are preferred by others, and social difficulties associated with autism spectrum disorder could be related to being less readable^40,41^. However facial expressivity was measured subjectively, making it unclear how it relates to readability and its associated likeability. An examination of how objectively measured facial expressivity makes one more readable could be key to understanding its social benefits, particularly in affiliative contexts where the costs of readability would be expected to be lower in comparison to competitive contexts.

While the face being a transparent reflection of motivations and states could be attractive for social partners, the ability to strategically modify one’s facial behaviour could also provide social benefits for the producer. This is related to the concept of social competence; “the ability of an individual to optimise its social behaviour depending on available social information”^42^, p. 679]. A measure of social competence could involve testing whether the individual can voluntarily exhibit behaviour that is effective in achieving a given social goal. Facial behaviour is likely to be particularly integral in achieving social goals, given its proposed role in social influence^18^. Yet, individual differences in the ability to use the face to achieve social goals has not been fully explored. Differences in facial control, in terms of being able to voluntarily produce and inhibit facial movement, could also impact this, and we know that people vary in their ability to control their facial muscles^43^. These capacities are likely to be important across a range of social contexts.

### Facial expressivity in competitive contexts

Competing interests are another common feature of human social interaction, and facial communication could have an important role in facilitating conflict resolution. There is a wealth of literature examining the factors that determine outcomes of bargaining games (e.g., the ‘ultimatum game’^44–46^). These are designed to examine what leads participants to reject or accept fair and unfair offers of monetary rewards from partners, and many studies show that communication strongly influences how people make these decisions^47–49^. These findings are relevant to real-life negotiations given that they typically involve extensive use of communication. Indeed the face may be particularly influential in negotiation due to its role in communicating intentions: Anger et al.,^7^ found that better recognition of facial expressions predicted better outcomes in a negotiation task. However few studies have examined how facial *production* affects one’s outcomes (although see^50^ for effect of ‘guilty’ facial movements on negotiation outcomes) and it is not clear whether being more or less expressive leads to better outcomes for the communicating individual. It would be reasonable to expect that keeping a still ‘poker face’ could be advantageous in order to avoid sharing information that could make one vulnerable^51,52^. However suppression of facial expression has been found to impede one’s own facial reading ability^53^ which carries social costs. Many conflicts are also not entirely antagonistic and people may be motivated to conserve the relationship or to take a more affiliative approach to resolving the conflict which can lead to better outcomes^52,54^. The face may be useful to communicate these intentions, and even communication of antagonistic intentions carries costs but also benefits^52,55^.

### Current study

Here we provide a large-scale systematic exploration of variation in facial behaviour as it emerges in typical social interaction. We aimed to test whether facial expressivity can be considered a stable individual difference and measure its social correlates and consequences. We measured facial expressivity in participants across varying contexts, with different social partners, and across different time points. Demonstrating consistency in an individual’s facial behaviour across these domains provides clear evidence that it is a stable individual trait. The social benefits of variation in behaviour may then be more likely to be long-term, and can be used to gain insight into its social function. In Study 1, we aimed to document the range of individual differences in facial expressivity within a semi-structured naturalistic social interaction across a range of contexts, including affiliation and conflict. We examined the stability of facial expressivity across different behavioural contexts, and how it relates to affiliative and competitive outcomes, as well as personality. In Study 2, we aimed to replicate our main findings in a larger dataset of unstructured social interactions, and additionally examined the stability of facial behaviour across multiple social partners and across time. Together, our findings will contribute to our understanding of the function of facial behaviour.

## Study 1

A table summarising all parts, measures and analyses of Studies 1 and 2 is provided in SI 1. Study 1 comprised three parts. The first part primarily involved a video call between the participant and a confederate posing as a participant, and was designed to elicit a naturalistic interaction across a range of typical behavioural contexts. This allowed us to examine individual variation in participants’ facial behaviour and test whether this behaviour varies depending on context. While the majority of the interaction was unscripted and free-flowing it was also semi-structured to create a variety of contexts. This allowed us to capture a more complete account of participants’ facial behaviour while still maintaining some degree of consistency across participants (e.g., same contexts and social partner). We chose the contexts to approximate typical social situations that occur in everyday life, rather than unusual or rare events. Participants also performed facial control tasks and completed self-report measures (e.g., personality). The second part involved the same participants uploading videos of themselves attempting to perform certain social goals, which adds a less naturalistic but more controlled experimental complement to the first part. The third part involved third party observers rating videos of the video call attendees, such as on likeability, how easy they were to read and how well they communicated their social goals. As such we were able to examine the social outcomes of facial behaviour based on the interaction and direct social partner (i.e., the confederate), as well as third party observers. Together we aimed to capture variation in a range of different facial behaviours and abilities, to examine how expressivity varies across typical contexts and to examine how they relate to social outcomes and correlates.

### Methods

#### Participants

All participants were recruited via an online recruitment platform (Prolific; http://www.prolific.co) and were compensated for their time.

##### Actors (Video call attendees)

Twenty participants were recruited for two pilot studies and 52 participants were recruited for the main study (24 men, 27 women, 1 undisclosed gender, mean age = 28.69 ± 9.27 years, of 19 nationalities; see SI 3 for further details), which involved taking part in a video call (using Zoom software; https://zoom.us/), and completing a series of questionnaires. Participants were told they would be compensated £5 with the possibility of a £3 bonus depending on performance in tasks (which they in fact all received), and that the purpose of the study was to measure and understand how people differ in aspects of communication during social interaction (see consent and debriefing forms; SI 1). Thirty-four of the original 52 participants participated in follow-up tasks which involved recording and uploading video clips of their face and were compensated an additional £5.

##### Raters

One hundred and seventy-six participants (87 male, 86 female, four undisclosed gender, mean age = 28.25 ± 8.92, of 33 nationalities; see SI 1 for further details, excluding six individuals who failed attention checks; see *Part 3—Ratings*) were recruited to take part in a video rating task, and were compensated £4.88 (see consent and debriefing forms; SI 1). The sample size was determined by aiming for 30 ratings per video which is in line with previous similar rating studies^56–58^.

#### Procedure

##### Pilot studies

We conducted two pilot studies to assess feasibility and to optimise the study design prior to data collection. Both pilot studies included feedback from participants. The first pilot study (N = 10) involved two participants interacting with each other. This was to assess how two genuine participants would interact in the context of the experiment to allow a confederate to emulate a similar interaction in the future studies. The second pilot study (N = 10) included a confederate posing as a participant. This was to trial different components of the call (e.g., amount of reward to offer, selection of joke) and to ensure a successful deception.

##### Part 1—Video call

The video call participants (actors) were directed from Prolific to an online survey platform (Qualtrics; http://www.qualtrics.com) to complete the consent form and, once completed, they received instructions to participate in a recorded video call using Zoom software (Zoom; https://zoom.us/; see Zoom call invitation, SI 1). They were told they would be interacting with another participant, but were instead interacting with a confederate (female, age 29, of Irish nationality), who was also simultaneously using the written chat to communicate as a researcher to both ‘participants’ (written from a third Zoom account, named ‘Researcher’).

**Figure 1:**
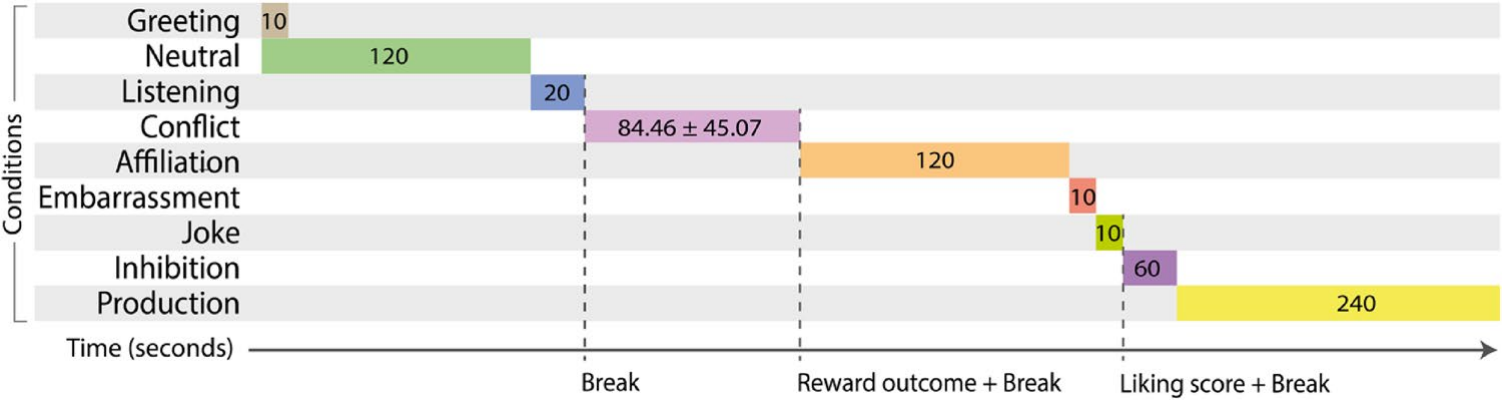
Timeline of conditions and outcomes of the video call. All numbers are time in seconds.

**Figure 2:**
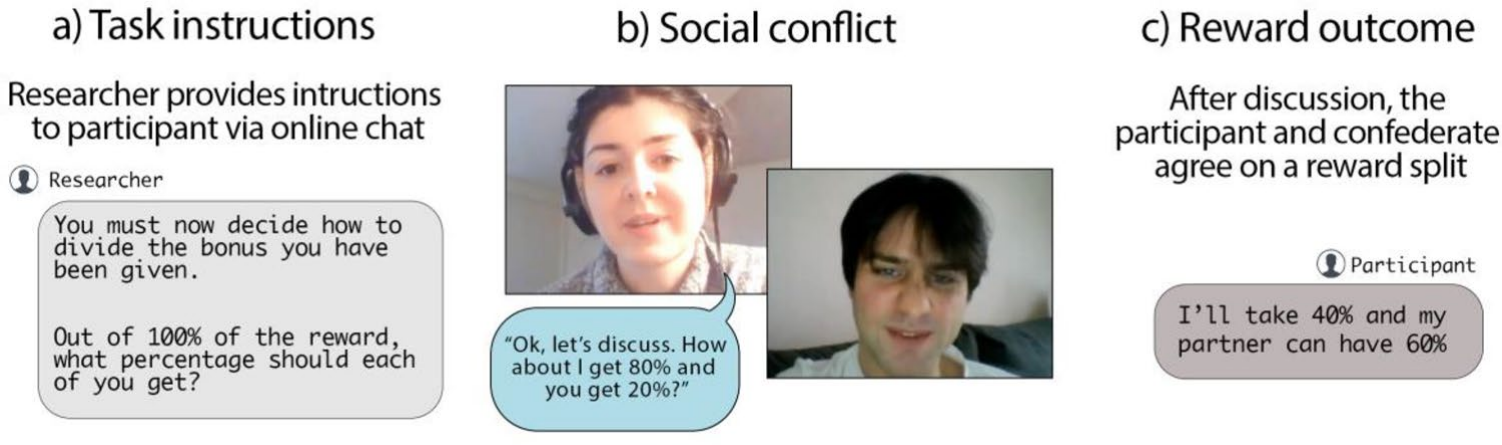
Excerpt of video call paradigm, taken from the conflict condition, during which the confederate suggests an unfair division of the reward, and both partners negotiate.

The video call involved nine conditions, which included three main naturalistic contexts; neutral, conflict and affiliation conditions. There were also two outcomes recorded; a liking score and reward outcome. The order of conditions and outcomes in the call and corresponding durations are outlined in Fig. 1, and a screenshot of the experimental set-up is provided in Fig. 2.

The participant and the confederate had visual and audible access to each other during each condition, and at the breaks they were asked via written chat to turn their videos and microphones off while they receive instructions for the next section. See video call script, SI 1, for details on instructions given to participants via written chat, and other logistical information such as when microphone and video are turned on and off.

##### Greeting condition

The first 10 s during which the participant and the confederate greeted each other was classed as the ‘greeting condition’. To maintain consistency, the confederate always waited for the participant to initiate verbal behaviour before responding.

##### Neutral Condition (Main context)

The neutral condition was designed to elicit a naturalistic initial acquaintanceship between two strangers. It lasted 2 min, during which the participant was instructed via written text to “*just get to know your partner. For example, you could talk about your experiences with using Prolific”.* The participant and the confederate interacted freely during this time.

##### Listening condition

At the end of 2 min, the confederate spoke about a topic relevant to the point in the conversation (e.g., how the pandemic has affected her work) for 20 s without stopping, in order to record facial behaviours related to listening.

##### Conflict Condition (Main context)

The conflict condition was designed to elicit a conflict of interest between the participant and the confederate. The participant was instructed via written text that they would be discussing with their partner how to divide a bonus reward. They were given no basis on which this should be divided. At the outset of the discussion, the confederate states:

*“Ok, let’s discuss. How about I get 80% and you get 20%?”*

The participant and the confederate then negotiated until they came to an agreement about how they should split the reward. In order to maintain some consistency across participants, the reasons given by the confederate for requesting a larger amount were restricted to four options, which were designed to appear unfair. The confederate gave reasons only when asked by the participant, and gave multiple reasons if asked multiple times. The four options were as follows;

*“I’d like to get as much of the reward as possible.”*

*“I think I did more of the talking in the first round, so deserve a higher amount of the reward.”*

*“I’d just like a higher amount.”*

*“I’m just trying to think of reasons why I should get a higher amount.”*

The primary motivation of the confederate was to elicit a conflict by proposing an unfair offer, and she agreed with a proposed reward division (thus ending the condition) once she believed the participant was unlikely to accept a lower amount (intending to mimic a naturalistic negotiation).

To preserve the relationship between the participant and the confederate following this conflict of interest, the participant received written communication from the researcher (via the chat, while participant and confederate were in visual and audible contact with one another) that the confederate had been given a secret task to try to receive a higher amount, as this amount would subsequently be allotted to both of them and would therefore benefit them both. The confederate then re-iterated this verbally and apologised.

##### Reward outcome

The percentage of the reward that the participant and confederate agreed would be allocated to the participant was extracted as a social outcome measure.

##### Affiliation Condition (main context)

The affiliation condition was designed to motivate the participant to make a good impression on the confederate. Following the conflict condition, the participant received written instructions (from the researcher) that their partner (the confederate) would be rating them on likeability, and that a higher rating would result in a higher bonus reward. The participant and confederate then continued to chat for 2 min (similar to the neutral condition, but with the extra motivation to be likeable).

##### Embarrassment condition

The confederate muted her microphone but continued to speak for 15 s (ignoring any attempts from the participant to inform her that her microphone was muted). She then unmuted her microphone, and stated *‘Oh I’m so sorry, I’m so embarrassed!’.* The 10 s following this statement was labelled the embarrassment condition. The aim of the embarrassment condition was to capture the participant’s reaction to an embarrassed social partner.

##### Joke condition

After the embarrassment condition, the confederate received written instructions from the researcher (while in visual and audible contact with the participant) to tell a joke. The confederate then told the joke “*How do you make a piece of paper dance? You put a little dance in it”.* The aim of the joke condition was to capture the participant’s reaction to a bad/nonsensical joke.

##### Liking outcome

After the joke, the participant and confederate went off camera, and the participant was asked privately by the researcher via written chat to rate their partner on a liking scale of 1–10. The confederate also privately rated how much she liked the participant on a scale of 1–10, which we used as a liking outcome. The confederate attempted to give an honest assessment of their impression of the participant based on their interaction.

##### Inhibition condition

The aim of the inhibition condition was to capture participants’ ability to inhibit facial expression. The participant received written instructions to try to keep a still face while their partner tried to make them move their face. The inhibition condition then commenced, during which the confederate first brought her face close to the camera and smiled for 15 s (creating an awkward social situation), and then afterwards read the following script (these measures were established through the pilot study as effective in causing difficulty for participants to keep a still face):

*“Now I have something to tell you while you try to keep your face straight. I am not a participant in this study. I have been posing as a participant but I am in fact a researcher in this study. The truth is, I intentionally turned my microphone off while continuing to speak to see how you would react, and I intentionally told a joke that was not funny to see if you would still laugh. The monetary bonuses you receive are not dependent on any performance in the tasks; you will be given the maximum possible bonus, and so will receive the maximum payment upon completion of the entire study. The task is now over and you can relax your face.”*

Participants were then debriefed about the study (see SI 1) and were asked if the deception caused any distress (no distress was reported by any participant).

##### Production condition

After debriefing, the final condition was the production condition, which aimed to capture participants’ ability to control their production of facial movements. Participants were shown moving images (GIFs) of 20 Facial Action Coding System^59^ action units, and asked to recreate the movements in isolation. GIFs were taken from iMotions website (https://imotions.com/blog/facial-action-coding-system/). Each were shown for 7 s, with a 5 s inter-trial interval displaying the instruction to “*Relax your face entirely”.*

##### Questionnaire measures

Following the Zoom call, participants were directed to Qualtrics to complete questionnaire measures. Participants were first asked whether they had any medical condition that affects the way their face moves (no participant reported a condition). They were then asked to indicate their agreement to statements about the video call they just participated in on a scale of 1–5 (*strongly disagree* to *strongly agree*). Some of these were to understand their intentions at particular parts of the call (see Table 2; ‘matching statement for actors’ column; responses compared with raters’ responses to calculate a ‘readability score’; see *Calculating Measures*). Others were used as manipulation checks to ensure the deception and elicitation of conditions were successful (see *Results—Manipulation Checks*).

Participants completed Gosling et al.’s^60^ brief 10-item questionnaire measuring the Big Five personality dimensions. This shorter inventory was used to minimise study fatigue given the time constraints after the long video call and other questionnaire measures, and because it has adequate psychometric properties even if diminished somewhat in comparison to longer instruments. Importantly, it has good test–retest reliability (r = 0.72), which is relevant to our aim of establishing whether facial expressivity correlates with stable traits. Whether it measures global or narrow personality traits as hinted at by^61^ is of less concern to our aims, in particular given that the majority of commonly-used scales appear to be facets of the Big Five scale^62^.

##### Part 2—Elicited social tasks

The same participants (actors) were contacted as a follow up approximately 6 months after the initial study to upload short video clips of their face while trying to achieve certain social goals (N = 34 agreed). These clips were chosen to reflect a range of behavioural contexts and goals which may occur during typical social interaction, for which use of the face could be feasibly beneficial. Participants were asked to record five video clips face-on, providing a clear image of their face, with nothing covering their face, in good lighting with the light source in front of them, and without the camera shaking. They were instructed to ‘*Please try to make the videos as natural a reflection of your own behaviour as possible. You will receive payment if the videos are uploaded correctly, and without any obvious deviations from natural delivery, e.g., laughing inappropriately.’*

In each video, they were asked to communicate one of five social goals; (i) Look friendly in a greeting, (ii) appear to be listening, (iii) reassure an embarrassed friend, (iv) appear threatening, and (v) disagree without being disliked (see SI 1 for full instructions and example video clips).

##### Part 3—Ratings

We presented the raters with clips from the video call (with the confederate cropped out) as well as clips uploaded by the actors, and asked them to rate them on various aspects via Qualtrics. From each participating actor we extracted 5 clips from the video call, and 5 uploaded video clips. All videos were muted to ensure ratings were based on visual rather than auditory cues. Each rater was presented with one video call clip and one uploaded video clip from each actor (randomly selected). All video call clips were presented first to raters in a random order, followed by the uploaded video clips which were also in a random order. A short explanation was given prior to each clip, and questions related to the clip followed its viewing. Raters were asked to respond to all questions on a sliding bar from 0 to 100, from *Not at all* to *A great deal.* Tables 1 and 2 indicate the accompanying explanation and questions specific to the video clip types (along with matching self-report statements about video call—see *Readability* section). For all video call clips, raters were also asked;

*As a first impression how much did you like this person?*

*How well do you feel you can read this person (i.e., have an idea of what is on their mind, what their intentions/motivations are)?*

**Table 1:** Clips from video call presented to raters.

Context of video clip	Description for raters	Question for raters	Matching measure of self-report from actors
Conflict (first 10 s)	The person's partner has just suggested that they share a monetary reward unequally, leaving the person with just 20% of the reward	Do you think this person disapproves of their offer?	I disapproved of my partner suggesting the reward be divided unevenly
		Is this person finding the conversation difficult?	I found the discussion about splitting the reward difficult
Affiliation (first 10 s)	The person is getting to know their partner	Is this person trying to make a good impression?	I was motivated to make a good impression after being told my partner would rate me on likeability
		Is this person trying to be polite?	I was motivated to be polite after being told my partner would rate me on likeability
Listening (first 10 s)	The person's partner is currently speaking to them about a topic	Does this person look like they are listening?	When my partner was speaking at length, I was listening
Joke (first 5 s)	The person’s partner has just told them a joke	Does this person find the joke funny?	I found my partner's joke funny ("How do you make a piece of paper dance? You put a little dance in it")
Embarrassment (first 5 s)	The person's partner appears to be very embarrassed about a mistake they made	Does this person feel sorry for their partner?	When my partner seemed embarrassed that her microphone was muted: I felt sorry for her
		Is this person trying to reassure their partner?	When my partner seemed embarrassed that her microphone was muted: I tried to reassure her

The ‘matching self-report of actors’ refers to statements about the video call to which the actors were asked to indicate their agreement. The correspondence between the raters’ responses to questions and the actors responses to matching statements was used to calculate the readability score (see *Calculating Measures*).

**Table 2:** Video clips uploaded by actors, presented to raters.

Actors’ social goal	Description for raters	Question for raters
Look friendly	The person is performing a greeting (saying "Hello, how are you?")	Does this person look friendly?
Appear to be listening	The person is reacting to a friend who is speaking	Does this person look like they are listening?
Try to reassure	The person is speaking to a friend who is embarrassed (saying "Don't be embarrassed, it's ok")	Do you think this person looks reassuring?
Look threatening	The person is disagreeing with a friend (saying "No, I don't agree with that")	Does this person look threatening?
Be liked despite disagreement	The person is disagreeing with a friend (saying "No, I don't agree with that")	Do you like this person?

The raters’ responses to the questions were used to calculate the actors’ ‘competence score’ (see *Calculating Measures).*

We included 16 attention checks to improve data quality. Raters were asked to select the context of the video clip they had just watched from 6 options, based on the explanation given prior to watching the video (they were unable to navigate back to the explanation). The resulting sample includes only those who answered correctly to more than 70% of the attention checks (6 were excluded). The resulting mean number of raters per video clip was 31.32 (SD: 1.41).

#### Data analysis

##### Measures

Where possible, (see SI 4 for details of missing data) for each actor we calculated (i) a facial expressivity score (N = 51), (ii) an inhibition score (N = 50), (iii) a production score (N = 51), (iv) a readability score (N = 47) and (v) a competence score (N = 33), as measures of their facial behaviour and abilities. We used the results from Gosling et al.’s^60^ inventory to measure their scores on the big five personality traits (openness, conscientiousness, extraversion, agreeableness, emotional stability). We also used the like scores from the confederate and raters, and the amount of the reward they secured in the conflict condition, as social outcome measures. Below details how these measures were calculated.

##### Facial expressivity score

We calculated a facial expressivity score for each participant, to quantify individuals’ facial behaviour during the video call. Facial movement was coded using iMotions^63^, an automated facial movement detection software based on the movements identified in the Facial Action Coding System; FACS^59,64^). FACS is an objective and quantitative approach to measuring facial movement based on underlying musculature, using ‘action units’ (AUs) as the units of measurement. This is the gold standard in measuring facial behaviour, as it avoids subjective impressions of facial expression. iMotions software integrates Emotient’s FACET technology (www.imotions.com/emotient), and allowed for extraction of sixteen action units (AUs 1, 2, 4, 5, 6, 7, 9, 10, 12, 14, 15, 17, 18, 20, 24, 28; excluding AU25, AU26 and AU43 as they couldn’t be differentiated from speaking or blinking, and ‘Smirk’ as it is an asymmetrical AU12) and seven expressions formed by combinations of action units which are traditionally considered to communicate ‘basic emotions’: ‘Happiness’ (AUs 6 + 12), ‘Sadness’ (AUs 1 + 4 + 15), ‘Surprise’ (AUs 1 + 2 + 5 + 26), ‘Fear’ (AUs 1 + 2 + 4 + 5 + 7 + 20 + 26), ‘Anger’ (AUs 4 + 5 + 7 + 23), ‘Disgust’ (AUs 9 + 15 + 16), ‘Contempt’ (AUs 12 + asymmetrical 14). From these, we extracted six measures in an exploratory attempt to capture facial expressivity (see Table 3).

**Table 3:** FACS Measures used to produce expressivity score.

Measure	Context-specific calculations (neutral, affiliation and conflict)	Overall calculation
Diversity Score	Measure of the N of unique AUs and how evenly they are represented. Calculated per condition using the following formula from^65^ $D_{n\tau } = \frac{{{\text{e}}^{{H_{n} }} }}{{\tau_{n} }} = \frac{{e^{ - } \sum\nolimits_{i = 1}^{S} {p_{i} \log (p_{i} )} }}{{\tau_{n} }}$ See SI 7 for further explanation of this formula	Mean diversity score across the three conditions
Rate	The N of AUs produced per minute in each condition	The mean of rates across the three conditions
Duration	The percentage time each AU was produced in each of the neutral, affiliation and conflict condition was first calculated. The sum of values from all AUs was calculated per condition	The mean of durations across the three conditions
AU Repertoire	The total number of unique AUs produced in the first 20 s of each condition	The total number of unique AUs produced in the first 20 s of the neutral, affiliation, conflict and listening conditions combined (80 s total)
Corrected AU Repertoire	The total number of unique AUs produced out of the first 25 AUs produced in each condition	The total number of unique AUs produced out of the first 25 AUs produced in the neutral, affiliation and conflict conditions combined (75 AUs total)
Combination Repertoire	The total N of unique AU combinations (i.e., simultaneous production of 2 or more AUs) in the first 20 s of each condition	The total number of unique AU combinations produced in the first 20 s of the neutral, affiliation, conflict and listening conditions combined (80 s total)

To create facial expressivity scores for each participant, we first performed four principal components analyses (PCA) on the six measures using the psych package^66^ in *R*^67^. In the first PCA we used the overall calculation of the six measures across contexts as outlined in Table 3, and in the other three we restricted calculation of the measures to each of the three main contexts: neutral, conflict and affiliation. SI 5 provides details of the PCAs. In all four PCAs, a single component emerged with all six measures loading moderately-highly. We therefore created a composite facial expressivity score by first transforming each expressivity measure to a Z-score distribution, and then calculating their mean score of the six measures. We also computed context-specific expressivity scores for each of the three main contexts; neutral, affiliation and conflict.

We additionally extracted the percentage of time in which the participants used each of seven facial configurations of emotion (averaged across the three main contexts), in order to quantify the use of these frequently-studied categorical expressions within typical social interaction, whether they are consistent with expected emotional states and the degree to which they explain variation in facial behaviour. iMotions estimates increase or decrease likelihood of each emotion based on the presence or absence of relevant action units, which are weighted according to their algorithm^68^ (see also SI 6).

#### Facial control (inhibition and production)

We include two measures of facial control; participants’ ability to inhibit facial movement in the inhibition condition, and their ability to produce facial movements in isolation in the production condition.

##### Inhibition score

Participants’ inhibition score was calculated by summing the percentage of time in the inhibition condition in which the participant was producing each AU, and taking the inverse of this value, such that a higher score indicated better facial inhibitory control.

##### Production score

A production score was calculated by attributing one point per trial in the production condition in which participants produced the appropriate AU in isolation, i.e., not accompanied by any of the other sixteen AUs coded by iMotions. As iMotions could only code 15 of the 20 AUs presented to participants, participants could get a maximum production score of 15, with a higher score indicating better voluntary production control.

#### Readability

##### Readability score

For each actor, we calculated a ‘readability score’ in an attempt to capture the tendency for individuals’ facial expression to be an honest and transparent reflection of self-reported motivations and states (as remembered by participants following the interaction, although we acknowledge this may not be fully accurate). To assess how well actors could be ‘read’ by raters, we used the actors’ agreement with statements related to the video call and compared them to raters’ ratings of the corresponding clip in the video call. For instance, we considered an actor to be more readable if they indicated that they found the joke funny in the video call, and raters also judged them as finding the joke funny. Table 2 outlines the 8 questions raters responded to (based on clips of the actors in the video call) as well as the matching statements the actors’ responded to about the video call. To calculate each actor’s readability score, we used the correspondence between the actor’s and raters’ responses.

For each actor, we took the mean rating from all raters for each of the 8 questions. We calculated Z scores for each of these 8 mean values and Z scores of the actors’ responses to corresponding statements (standardised across all actors), in order to standardise these two initially different scales. We then found the absolute difference between the raters’ standardised ratings, and the actors’ standardised response to the corresponding statements, and summed these 8 difference values. As this resulting score indicated the discrepancy between the raters’ judgements and the actor’s actual intentions/motivations, we used the inverse of this value as the final readability score, in order to make the final score a positive indication of readability.

#### Perceived readability

We measured how readable raters believed the actors to be, as we thought this could potentially have a stronger influence on social outcomes than how readable they actually were. For each actor, we took the mean value of all raters’ responses to the question “*How well do you feel you can read this person (i.e., have an idea of what is on their mind, what their intentions/motivations are)?*” for each of the 5 video call clips. We used the mean of these 5 values to calculate each actor’s perceived readability score.

##### Competence score

We calculated a ‘competence score’ per actor to measure how well they were able to intentionally communicate a message with their facial behaviour. This is related to, but separate from, the readability score and the production score. A more readable individual may or may not intend to communicate their internal motivations, and an individual with higher voluntary production control may or may not be able to produce the appropriate facial movements at the right time to achieve their social goals. For this reason we measured participants’ abilities to communicate social goals with their face based on their uploaded video clips as assessed by the raters. Table 2 outlines the social goals the actors attempted to achieve in their video clips, and the corresponding questions the raters responded to upon viewing the clips. For each actor, we took the mean rating of each clip, and calculated competence score from the mean of these 5 values.

#### Social outcomes

##### Confederate’s like score

We measured the degree to which the confederate liked each actor. Despite being subjective, we felt that this was a relevant social outcome as the confederate was a social partner interacting directly with the actor, and was naïve to this hypothesis at the time of the study. The confederate’s judgement approximates an ecologically valid indicator of rapport that emerged as a result of the actor’s behaviour within social interactions. We recorded the confederate’s liking score prior to her revealing that she was a confederate (therefore encompassing the overall impression given by the actor from all preceding conditions: neutral, listening, conflict, affiliation, embarrassment and joke conditions).

##### Raters’ like score

We measured the degree to which third party observers (the raters) liked each actor. This gives an indication of the likeability of the actors from a wider sample of people. For each actor, we took the mean value of all raters’ responses to the question “*As a first impression how much did you like this person*?” for each of the 5 video call clips. We used the mean of these 5 values to calculate a raters’ like score for each actor.

##### Reward outcome

We measured the amount of reward negotiated by the actors in the conflict condition. This is reflective of the outcomes of conflicts of interest that are typical in social interaction. As 46.94% secured half of the reward, and 53.06% secured less than half of the reward (ranging 20–49% of the reward), we transformed the reward outcome into a binary variable, whereby actors’ reward outcome was either scored as ‘High’ (achieved half), or ‘Low’ (achieved less than half).

#### Exploratory analyses

We conducted a number of exploratory analyses to examine how facial behaviour of the participants (actors) in the video call varied across individuals and contexts, and how it related to social correlates and outcomes. Due to the exploratory nature of this first study, we did not control for multiple tests, as appropriate multiple test adjustments are difficult or impossible to ascertain in exploratory studies without causing disproportionate risk of type 2 error^69^. We conducted all tests in *R*^67^.

We performed six repeated-measures analyses of variance (ANOVA) using the *aov* function in R to test whether actors’ facial expressivity differed significantly across the three main video call contexts; neutral, affiliation and conflict (we looked at each facial expressivity measure separately, as calculation of expressivity scores involved standardising measures within each context, thus reducing variation). We also performed a repeated-measures ANOVA to test whether the use of the seven emotional configurations differed across these three contexts.

We computed Pearson’s correlation coefficents using the *cor.test* function in R to assess the relationship between (i) facial expressivity scores across conditions, (ii) the six facial measures: facial expressivity score, inhibition score, production score, competence scores, readability score, perceived readability, (iii) the five personality scores and the six facial measures, (iv) the confederate’s and raters’ like scores and (v) both like scores and the five facial measures.

Finally, we performed a generalised linear model (GLM) using the *glm* function in R and fit with a binomial distribution, to assess the interaction between personality and expressivity in predicting reward outcome. The purpose of this exploratory test was to identify whether facial behaviour was associated with greater benefits for those of certain personality traits, which would provide preliminary support for the hypothesis that the benefits of facial expressivity vary depending on one’s social niche.

### Results and discussion

#### Manipulation checks

Participants’ self-report responses to statements about the video call indicated that the protocol successfully elicited a naturalistic social interaction, including both the affiliative and conflict conditions. No participant strongly agreed that they knew the participant was a confederate, and just 14.29% somewhat agreed. Detailed information regarding the outcome of the manipulation checks can be found in the supplementary materials (see SI 2).

#### Is facial expressivity stable across behavioural contexts?

Our data revealed considerable and stable individual variation in facial expressivity (see Fig. 6). There were moderate-to-strong correlations between facial expressivity scores in the three main naturalistic conditions (Neutral and Affiliation: r(49) = 0.82, *p* < 0.001; Neutral and Conflict: r(46) = 0.61, *p* < 0.001; Affiliation and Conflict: r(46) = 0.59*, p* < 0.001), and repeated measures ANOVAs indicated that there was no significant difference in measures of facial expressivity across these conditions (see SI 8 for model statistics; note that an initial model with a full sample indicated that AU rate differed significantly across contexts, however when we removed influential datapoints identified in boxplots the model was no longer significant). This indicates there is no evidence that participant expressivity varied across conditions. This provides some evidence of facial expressivity being an individual trait, rather than varying primarily depending on situation. Additionally, repeated measures ANOVAs indicated that the use of each of the seven emotional configurations were produced similarly across contexts (See Fig. 7, see SI table 7b for model statistics), all at low frequency apart from ‘happiness’ (smiling). This demonstrates that even the types of expressions used vary more depending on the individual rather than the context.

Together, these analyses indicate that individual differences in facial and emotional expressivity appear to be stable across behavioural contexts.

#### How does facial expressivity relate to facial control, competence and readability?

**Figure 3:**
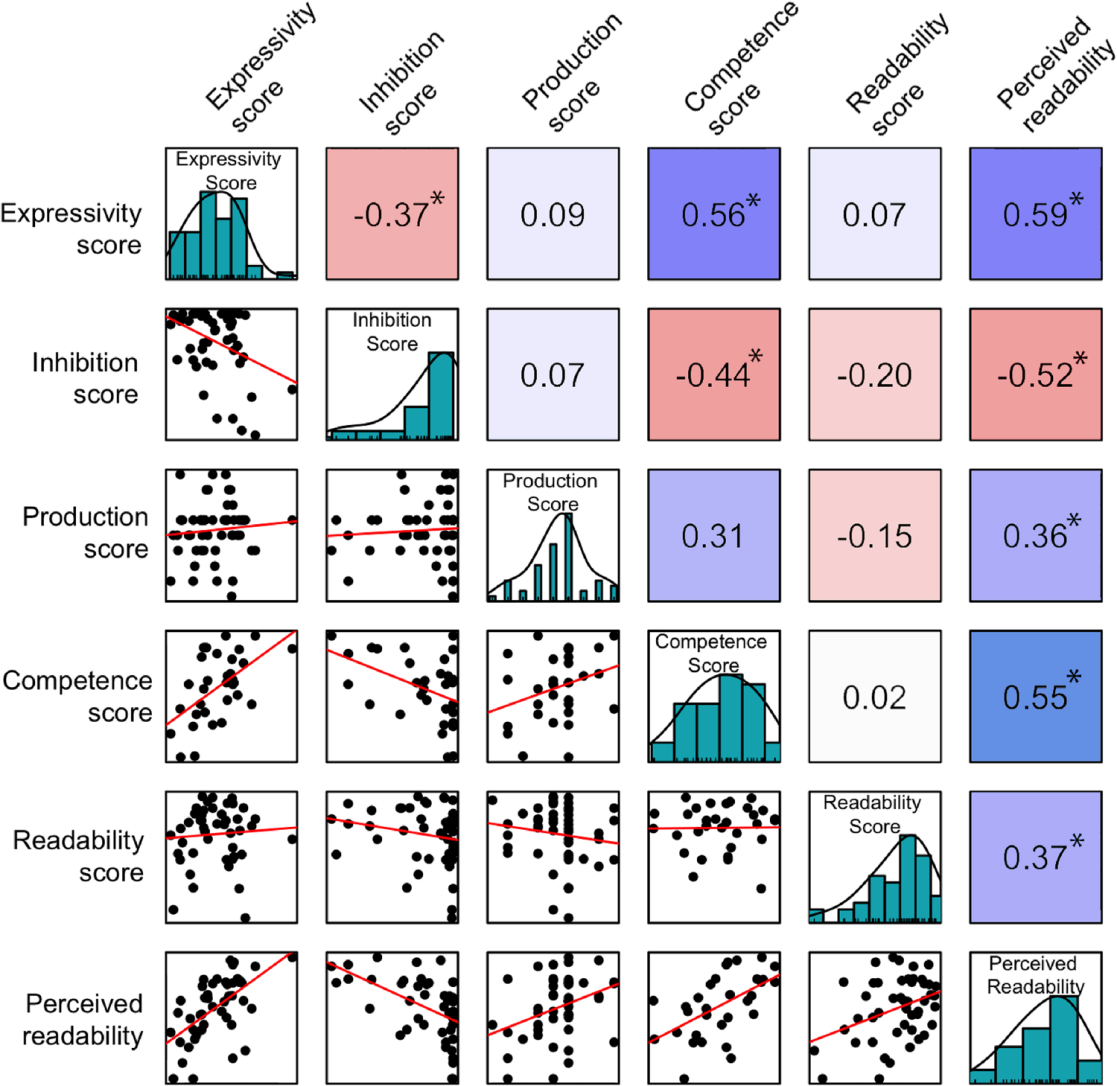
Correlations between expressivity and other facial measures. Numerical values indicate Pearson’s R. Blues represent positive correlations, reds represent negative correlations (with the luminosity representing the strength of association). *p < 0.05.

Figure 3 displays correlations between facial expressivity scores and other facial measures (inhibition, production, readability, perceived readability and competence), which range from weak to strong. More facially expressive individuals had poorer facial inhibitory control, which could indicate that some proportion of facial expressivity within the social interaction was a result of automatic facial reactions to the social interaction. Inhibition did not correlate with production score, so the ability to inhibit one’s facial movement, and to voluntarily produce facial movement may be distinct and independent aspects of facial control.

More facially expressive individuals had greater facial competence, which could indicate that the regular use of facial behaviour in every day interactions could be related to competent usage. It is notable that facial competence did not clearly relate to having better control over facial muscles, with more facially competent individuals in fact having a poorer ability to inhibit facial movement. This could suggest that there are a range of facial strategies that are suitable to a given social goal, and finer dexterity in muscular movement may be less important than the social cognitive ability to select an appropriate behavioural strategy. However, these findings should be interpreted with the caveat that facial competence and inhibition were measured using tasks that were more controlled but less naturalistic than the facial expressivity measures (e.g., participants may have used more exaggerated expressions in the social tasks than their typical behaviour). Future research should build on our findings by developing more ecologically valid methods of measuring facial competence and inhibition.

Surprisingly, more facially expressive participants were not more readable, though raters perceived them to be more readable. It could be that in first impressions we are actively searching for information about our social partner, and a facially expressive partner *appears* to be easier to understand simply because they are sending more information. Despite this, attending to specific facial configurations may have helped raters to accurately interpret actors’ motivations, thoughts and intentions, rather than their general trait of facial expressivity, but this remains an empirical question. Interestingly however, more readable people were also perceived as more readable. There are likely to be individual differences in the ability to read social partners, and better ‘readers’ could have driven this positive correlation. This variation in ability to read social partners appears to be predictive of social success^17^ and is a valuable area for future exploration.

#### Which personality types are more facially expressive?

Facial expressivity score had a small significant positive correlation with self-reported agreeableness (r(49) = 0.31, *p* = 0.025) but no other personality measure (see figure S2, also showing that personality does not correlate significantly with any other facial measure). This relationship was consistent across contexts, with more agreeable participants having higher facial expressivity scores in neutral (r(49) = 0.29, *p* = 0.04), affiliation (r(49) = 0.32, *p* = 0.02), and conflict contexts (r(46) = 0.36, *p* = 0.01). Given that agreeableness is a prosocial trait characterised by a motivation to foster smooth interpersonal relations^70–72^, the greater facial expressivity displayed by agreeable individuals could be a method used to build rapport with their partner.

#### Are more facially expressive people more liked by others?

The confederate’s liking score correlated significantly and moderately with the raters’ liking score (r(45) = 0.47, *p* < 0.001), indicating that third party impressions of likeability are similar to those of a social partner who was interacting directly with them.

**Figure 4:**
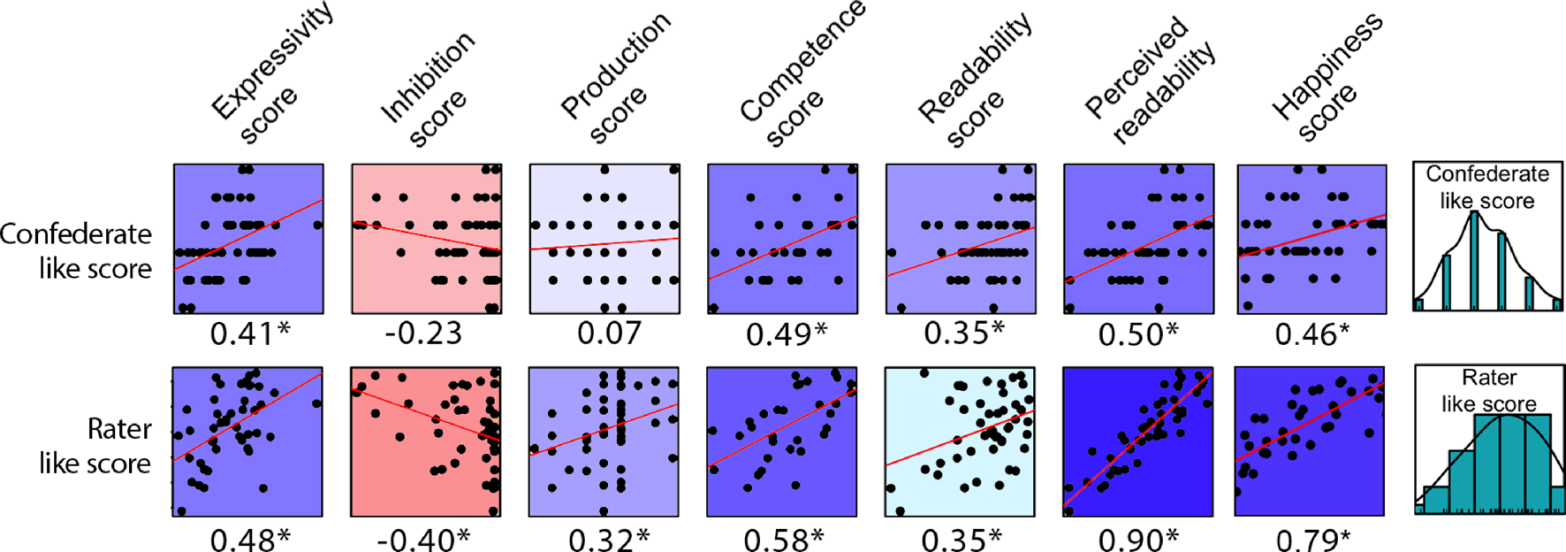
Correlations between both like scores and facial measures, with values indicating Pearson’s R. Blues represent positive correlations, reds represent negative correlations (with the luminosity representing the strength of association). *p < 0.05.

Correlations (ranging from weak to strong) between both liking scores (confederate’s and raters’) and facial measures are displayed in Fig. 4 (see SI 8 for sensitivity analyses). This shows that more expressive participants were liked more both by the confederate and by the raters, indicating that facial expressivity is a likeable trait. While we cannot rule out that the confederate liking a participant caused them to be more facially expressive, this expressivity resulted in being preferred by third party observers. This indicates that facial expressivity leading to greater liking is a plausible interpretation of the direction of causality. This may point towards an affiliative function of facial expressivity, providing some evidence of its role in creating positive social outcomes.

Participants who were more facially competent were also more liked by others, supporting our proposal that the ability to flexibly adjust facial behaviour to suit a given context could be a key component of social competence. This is in contrast to Fultz et al.’s^33^ finding that ‘performance expressivity’ (ability to accurately transmit an emotional and interpersonal message nonverbally) was unrelated to liking. This could be due to differences in the tasks used, with theirs requiring observers to identify the context of acted scenes, while ours required observers to identify the social goal of the participant. However participants with poorer facial inhibitory control, but better production control, were liked more by raters but not by the confederate. This is consistent with the suggestion that the ability to select an appropriate behavioural response within one’s repertoire is more important than muscular control within a social interaction. Greater facial expressivity could potentially mediate the relationship between poorer inhibition and raters’ liking. The costs of poor inhibitory control may have been more apparent within the social interaction, such as failing to inhibit a disapproving facial reaction, which could explain why the confederate did not prefer those with poorer inhibitory control.

Participants who were readable, and perceived as readable were more liked by others. This is consistent with findings that a more easily judged personality is a preferred social trait^38^, and supports the suggestion that being more predictable carries social benefits.

We note that personality traits did not predict being liked by raters, although the confederate showed a small significant preference for participants who were more extraverted (r(50) = 0.341, *p* = 0.013) and open (r(50) = 0.348, *p* = 0.011; see Figure S3). These suggest that personality correlates of facial behaviour did not drive its association with likeability. With respect to the emotional expressions, participants who produced the ‘happiness’ expression more were more well-liked both by the confederate (moderately; r(48) = 0.45, *p* < 0.001) and by the raters (strongly; r(43) = 0.75, *p* < 0.001), and those who produced the ‘disgust’ expression were less liked by the raters (weakly; r(43) = -0.31, *p* = 0.04). No association with like score was found with other emotional expressions (see figure S4).

Together, these show that facial expressivity, and other aspects of facial behaviour and ability, are related to being liked by others, whether these others are direct social partners or third-party observers.

#### Does facial expressivity relate to conflict resolution?

**Figure 5:**
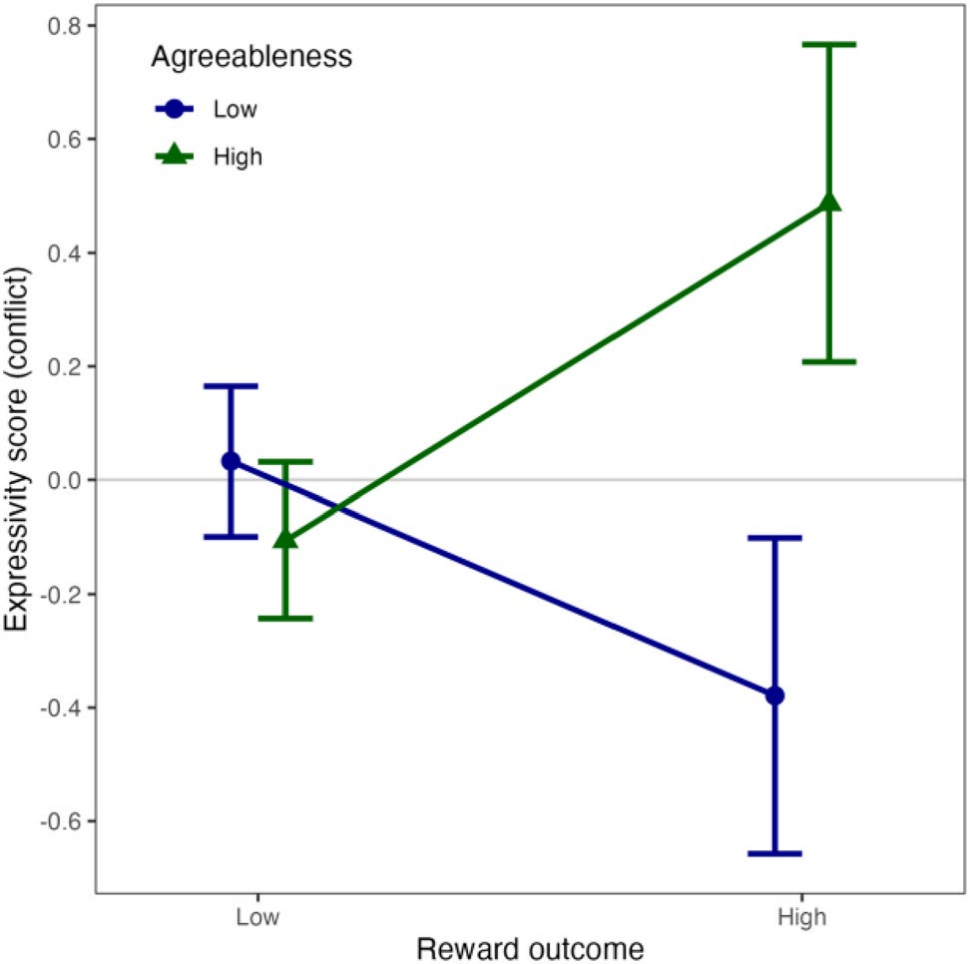
Interaction between facial expressivity and agreeableness in predicting reward outcome. Agreeableness divided into high and low based on median split for purpose of illustration. Error bars indicate standard error.

We performed a GLM to test whether agreeableness and facial expressivity in the conflict interacted to predict the reward outcome. The model shows a main effect of facial expressivity (ß = 8.963, SE = 3.711, *z* = 2.415, *p* = 0.016) but also a significant interaction between agreeableness and facial expressivity (ß = -1.838, SE = 0.741, *z* = -2.480, *p* = 0.013). This shows that more agreeable participants secured a higher proportion of the reward if they were more expressive in the conflict condition, but expressivity had no effect for less agreeable participants (see Fig. 5). This points towards facial expressivity being used as a tool to acquire tangible resources only for those tending towards a more agreeable ‘social role’ (i.e., the tactic an individual uses in response to social challenges^73^. This is consistent with social niche specialisation hypothesis, which provides an evolutionary explanation for why individuals do not behave flexibly in the most effective way to suit a given context, but rather show consistent differences in social behaviour^74^ to allow them to occupy and exploit a specific niche. It is also complementary to Schmidt & Cohn’s^75^ work which explored the adaptive role of facial expression, and emphasised the importance of examining the costs and benefits of facial expression in different socioecological contexts. Here, our findings point towards agreeableness and facial expressivity being tied together within a social niche, one that could be more cooperative or affiliative. While this finding should be interpreted with caution as it is highly exploratory and preliminary, we suggest that examination of varying costs and benefits of facial behaviour depending on personality or other social variables could be a fruitful avenue for future research.

## Study 2

Our main findings from Study 1 were evidence of stability of facial expressivity across behavioural contexts, and of its association with being liked and agreeableness. The purpose of Study 2 was to test whether these replicated in a larger dataset of unstructured naturalistic social interactions, and to examine other aspects of stability. The use of a single confederate in Study 1 allowed us to create the behavioural contexts and facilitate consistency of the social interaction across participants. Individual characteristics of the confederate that could likely influence the interaction (e.g., personality, age, gender, attractiveness) remained consistent, reducing noise in the data. While this allowed us to examine whether participants were similarly expressive across behavioural contexts, we could not test whether it varied depending on social partner. We also could not rule out that some of our findings were an artefact of interacting with this specific individual. The use of a confederate and some scripted interactions (although the interaction was largely naturalistic) may have limited the ecological validity to some extent. As participants only took part in one video-called interaction, we also could not test whether their expressivity remained consistent over longer time periods. Therefore, in study 2, we examined facial expressivity in pairs of real participants engaging in unstructured social interaction, and tested our main findings of interest; specifically, that facial expressivity is a stable trait related to being liked by others and personality. The larger sample size in study 2 also helped provide confidence in our findings, and to control for multiple variables in the same models rather than using exploratory correlations.

### Methods

#### Dataset

We used the CANDOR corpus (Conversation: A Naturalistic Dataset of Online Recording^76^) which is a dataset of 1656 recorded conversations between pairs of participants from the United States of America, with a total of 1456 participants, 51% of which took part in more than one conversation. The conversations were conducted via video call (using web application TokBox OpenTok Video API) and were entirely unstructured, lasting a minimum of 25 min [mean = 30.52, standard deviation = 7.74 min]. Participants also completed surveys, which included the extra-short form of Soto & John’s^77^ big five personality inventory, and a rating from 1 to 7 of how much they liked their conversation partner. These provided us with personality scores and a liking outcome equivalent to Study 1. Further details on the dataset can be found at Reece et al.^78^.

#### Data analysis

To reduce time taken to process videos, we measured participants’ facial expressivity based on the first five minutes of each conversation, and validated this by comparing these clips with expressivity measures in the full conversation in a subset (N = 152). In our final sample we excluded videos from participants in which iMotions software could not detect their face more than 10% of the time, resulting in 2756 conversations and 1315 unique participants. We extracted the same six facial expressivity measures using iMotions as in Study 1 (see Table 3).

As in Study 1, in order to calculate facial expressivity scores we first performed a PCA on the six facial expressivity measures from the five-minute video clips, using the mean values across all conversations for those participants who took part in more than one conversation. Unlike in Study 1, two components emerged from this PCA (see SI 5 for further details; we also report main results using individual facial expressivity measures to facilitate comparison between studies 1 and 2 in SI 8). We therefore calculated two facial expressivity scores per participant per conversation based on the measures that loaded highly on component 1 and component 2, respectively. As such, facial expressivity score 1 was calculated from the mean Z scores of rate, duration and combination repertoire, and facial expressivity score 2 was calculated from the mean Z scores of repertoire, controlled repertoire and diversity score. We additionally calculated use of emotional expressions as in Study 1.

While in Study 1 we examined stability of facial expressivity across contexts, here we examined stability across interactions with different social partners and across time. In those participants who participated in more than one conversation (N = 640), we tested whether their facial expressivity scores in each conversation correlated significantly with their mean scores across their other conversations. For those participants whose first and last conversations were at least 4 months apart (N = 115), we tested whether their facial expressivity scores correlated significantly at these two time points. For these we calculated Pearson’s r used the *cor.test* function in R, and controlled for multiple tests using Bonferonni’s correction^79^.

To test whether the relationship we identified in Study 1 between facial expressivity and personality replicated in Study 2, we constructed two linear mixed models using the *lmer* function from the *lmerTest* package^80^, and tested the significance of the fixed effects using the *summary* function. We included the five personality measures as fixed effects, the participant ID and partner ID as random effects, and the two facial expressivity scores as outcome variables. Marginal R^2^ for fixed effects was calculated using the *partR2* package^81^.

Finally, to test whether participants who were more facially expressive were more liked by their partner, we conducted an ordinal logistic regression using the *ordinal* package^82^, with the participant’s liking rating from their partner as the outcome variable, and both facial expressivity scores as fixed effects. To control for potential shared effects of personality and facial expressivity on liking rating, we included the three personality measures that predicted facial expressivity in the previous models as fixed effects (see results). Additionally, given the prevalence of the ‘happiness’ expression (see results) we included this as a fixed effect, to account for the possibility that any effect of facial expressivity on liking was accounted for by increased smiling. Variation inflation factors indicated no issues with multicollinearity in the predictor variables.

### Results and discussion

Facial expressivity in the 5-min clips correlated very strongly with expressivity in the full conversations (N = 152; facial expressivity score 1; r = 0.91, *p* < 0.001; expressivity score 2; r = 0.70, *p* < 0.001) indicating that the 5 min clips we used were a valid representation of expressivity in the full conversation.

#### Is facial expressivity stable across social partners and time?

**Figure 6:**
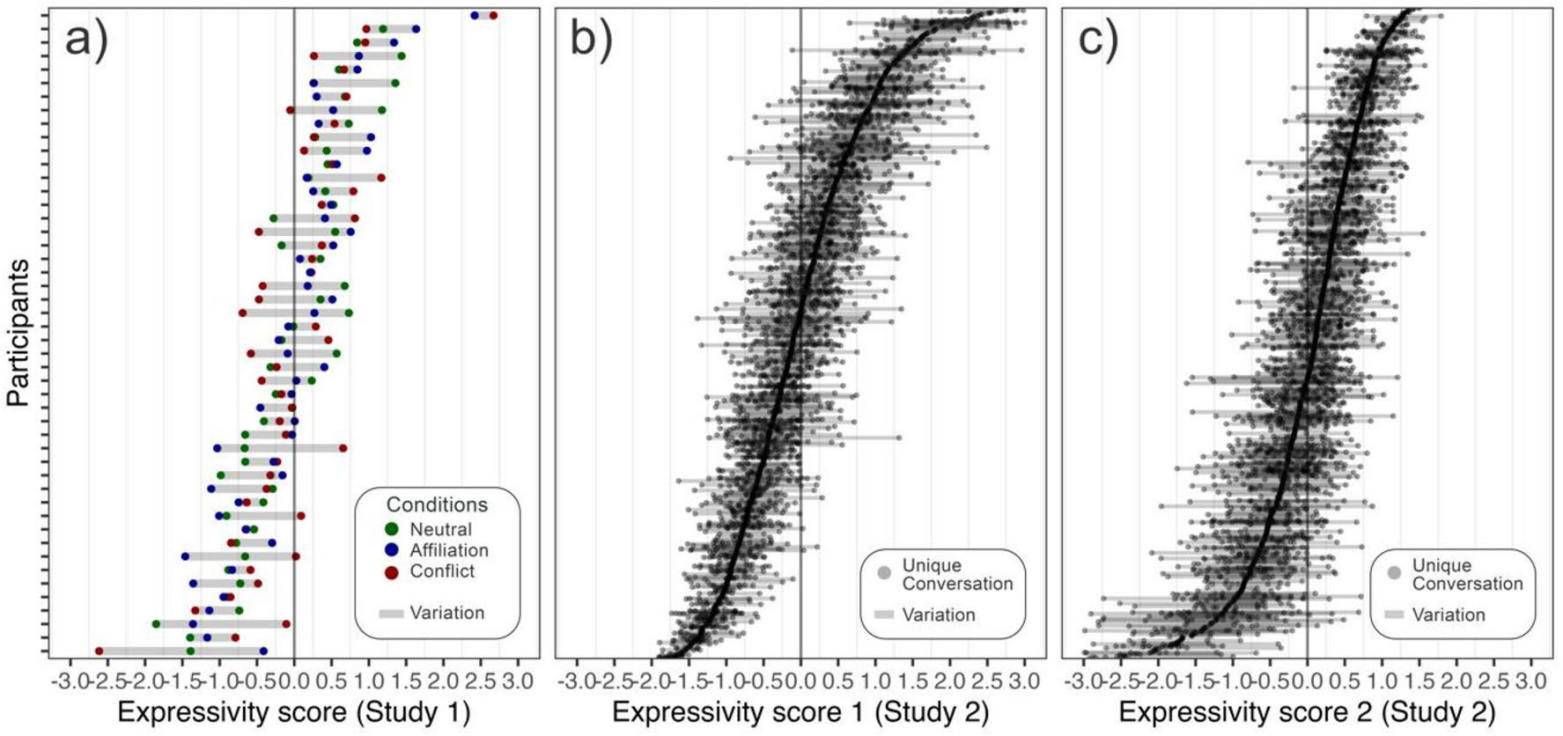
Variation in facial expressivity scores. (**a**) Expressivity scores across contexts from Study 1. (**b**) and (**c**) Expressivity score 1 and 2 across social partners in Study 2, respectively.

As illustrated in Fig. 6, we found that participants’ facial expressivity scores in a given conversation showed a strong positive significant correlation with their mean expressivity scores in all other conversations (facial expressivity score 1; r = 0.74, *p* < 0.001, facial expressivity score 2; r = 0.59, *p* < 0.001). This provides evidence that facial expressivity does not vary across social partners. Participants’ facial expressivity scores in their first and last conversation at least 4 months apart also correlated significantly although less strongly (facial expressivity score 1; r = 0.55, *p* < 0.001, facial expressivity score 2; r = 0.36, *p* < 0.001). This provides evidence that facial expressivity is stable across time. Together with our finding from Study 1 that facial expressivity is consistent across behavioural contexts, this provides a robust indicator that facial expressivity is a stable trait.

**Figure 7:**
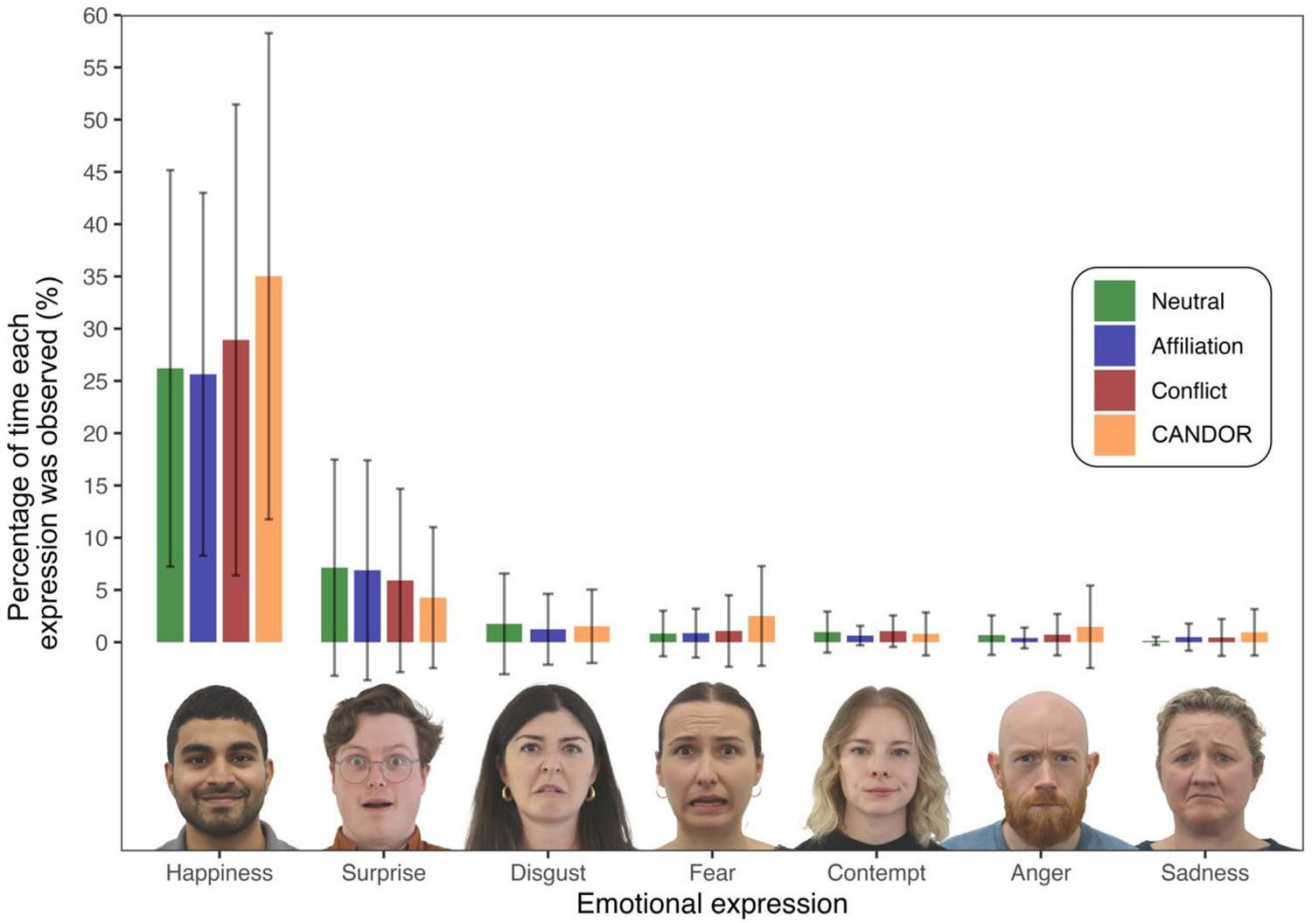
Use of emotional configurations across contexts, using the percentage of time each of the expressions were produced by the actor, in the neutral affiliation and conflict conditions (Study 1), and the CANDOR interactions (Study 2). Action units indicating increased likelihood of emotion by iMotions: Anger = AUs 4 + 5 + 7 + 23, Contempt = AUs 12 + asymmetrical 14, Disgust = AUs 9 + 15 + 16, Fear = AUs 1 + 2 + 4 + 5 + 7 + 20 + 26, Happiness = AUs 6 + 12, Sadness = AUs 1 + 4 + 15, Surprise = AUs 1 + 2 + 5 + 26.

We note that although participants were not observed in multiple contexts, their usage of emotional expressions showed a similar pattern to Study 1 (see Fig. 7). A mean of 39.97% [SD = 23.69] of participants’ facial usage was coded as ‘Happiness’, while all of the other emotional expressions combined comprised 9.87% [SD = 10.73] of participants facial usage.

#### Is facial expressivity related to agreeableness or other personality traits?

We replicated our finding from Study 1 that more agreeable participants were more facially expressive with respect to facial expressivity score 1, but not facial expressivity score 2 (see Table 4). Facial expressivity score 1 might be more related to frequency of facial movements as opposed to greater diversity of movements associated with score 2. More frequent facial signalling therefore appears to be an agreeable behaviour. A considerable proportion of variation in both expressivity scores are explained by the individual, again pointing towards the trait-like nature of facial behaviour, while the fixed effects explain a small but significant proportion of variation (see Table 4). Unlike Study 1 however, we found that more extraverted and neurotic participants were also more facially expressive with both scores. Indeed, of the personality variables, extraversion was the strongest predictor of facial expressivity. Given that extraverted individuals are also more talkative^83^ this suggests they may be more communicative in general. We speculate that the association between neuroticism and facial expressivity was related to social preoccupation, given Whitehouse et al.’s^17^ finding that more stressed participants were not more facially expressive in a non-social task. Therefore, our findings are consistent with the interpretation that facial communication may be used as a tool to build rapport.

**Table 4:** Results from two linear mixed models (both N = 2710) testing whether personality predicts facial expressivity, with participant’s expressivity score 1 and 2 in a given conversation as the outcome variables, respectively.

	Expressivity Score 1				Expressivity Score 2			
Random Effect	Variance Explained				Variance Explained			
Participant (N = 1290)	57.83%				31.83%			
Partner (N = 1318)	0.77%				0.37%			

Fixed Effect	Marginal R^2^	t	*p*		Marginal R^2^	t	*p*	
Agreeableness	0.002	2.337	0.019	*	< 0.001	− 0.081	0.935	
Extraversion	0.006	3.559	< .001	***	0.01	4.988	< .001	***
Openness	< 0.001	− 0.536	0.592		< 0.001	− 1.258	0.209	
Conscientiousness	< 0.001	0.242	0.809		< 0.001	− 0.393	0.695	
Neuroticism	0.003	2.651	0.008	**	0.002	1.977	0.048	*

*p < 0.05, **p < 0.01, ***p < 0.001.

#### Is facial expressivity a likeable trait?

Similarly, we replicated our finding that more facially expressive participants were more liked by their social partner with facial expressivity score 1, but not with score 2 (see Table 5; a 1 unit increase in Expressivity Score 1 (ranging from − 1.903 to + 3.970) was associated with a 0.158 increase in liking score (ranging from 1 to 7). Extraversion and increased smiling also predicted being liked more by their social partner. In this model we controlled for the three personality traits that were associated with facial expressivity, and still found that facial behaviour predicted being liked more strongly than personality. It is notable that we were able to replicate findings from study 1 related to liking and agreeableness with expressivity score 1, but not 2. It is possible that the frequent style of facial expressivity (score 1) is that which is associated with affiliative social behaviour and outcomes, while the more diverse expressivity style (score 2) has other potential unexplored benefits. Nonetheless, together our findings indicate that aspects of facial expressivity are strongly related to positive first impressions.

**Table 5:** Result from ordinal logistic regression (N = 2586) testing whether facial expressivity predicts liking, with partner’s liking score from a given social partner as the outcome variable.

Random Effect	Variance		Standard Deviation			
Participant (N = 1279)	0.388		0.623			
Partner (N = 1300)	1.017		1.001			

Predictor Variables	β	Standard error	*z*	*p*		Estimated Probability
Expressivity Score 1	0.158	0.07	2.271	0.023	*	0.539
Expressivity Score 2	− 0.082	0.068	− 1.204	0.228		
Agreeableness	0.072	0.061	1.194	0.233		
Extraversion	0.127	0.05	2.522	0.012	*	0.532
Neuroticism	0.073	0.045	1.656	0.1		
Happiness expression usage	1.06	0.244	4.364	< .001	***	0.744

*p < 0.05, **p < 0.01, ***p < 0.001.

## General discussion

We have provided a quantitative ethological assessment of variation in facial behaviour within semi-controlled and uncontrolled social interactions, as well as in elicited social tasks. Our results suggest that facial expressivity is a meaningful individual difference that is stable across contexts, social partners and over time, and is related to positive social outcomes and personality. More facially expressive people were more well-liked both by their social partner and by third party observers, and were found to be more agreeable in both studies, and more extraverted and neurotic in study 2. Moreover, participants who smiled more, were more facially competent, more readable (both objectively and subjectively), and who had better voluntary facial production control but poorer facial inhibitory control were more well-liked either by their social partner, third party observers, or both. Finally, more facially expressive people tended to negotiate a higher reward outcome, but only if they were also more agreeable. Together, our findings point towards an affiliative function of facial expressivity, with consequences that are likely to be important in a real-world social context.

The striking individual differences in facial behaviour we have identified, much of which is not captured by the prototypical configurations, emphasise the need to move away from universality as the dominant focus in facial expression research. Our data provide clear evidence that people vary in facial expressivity within their everyday social interactions and that this is a stable trait. It is necessary as a next step to measure variation in facial behaviour across different types of social relationships, such as between close friends, and across a wider range of contexts, such as in work. Nonetheless, we have uncovered ample inter-individual variation to facilitate understanding its social function in first impressions.

Our findings point towards facial behaviour serving a social bonding or relationship-management function. This is highlighted by the positive social outcomes associated with greater facial expressivity, and its relation to prosocial personality traits. The greater facial expressivity displayed by agreeable, extraverted and neurotic individuals could reflect an attempt to build rapport with their partner, and it appeared to be effective in doing so. Greater likeability can be considered a positive fitness consequence of facial expressivity supporting Schmidt & Cohn’s^75^ proposal that cooperation and affiliation are the primary functions of facial expression. Whether or not using facial expressivity as a tool to increase social bonding results in larger or stronger social networks is an important next step in assessing its fitness benefits. Facial expressivity could also be a tool for conflict resolution, given that it predicted a higher reward outcome for more agreeable people. However, we would argue that affiliation is the wider function, as affiliation is one approach to conflict resolution, and one that is more likely to be pursued by agreeable people^84,85^.

Our findings should be interpreted with the caveat that the majority of participants in our samples were of US American or European nationalities. It is important for future research to examine the cross-cultural generalisability of our findings, as facial expressivity may be associated with different outcomes in different cultural contexts. The concept of ‘display-rules’ is well-studied in emotion research, which is thought to be a set of social norms that guide the appropriateness of expressing emotions, and these norms differ cross-culturally^86^. Given increasing acknowledgement that facial expressivity does not necessarily equate to emotional expressivity e.g.,^21^, it is an open question whether or not social norms guide facial expressivity in social interaction, and whether facial expressivity is received differently across cultures. Large-scale cross-cultural studies will be invaluable for generating nuanced hypotheses and robust evidence of the function of facial behaviour.

The question remains as to why such striking variation is maintained in the population. This is a challenging question that likely requires a new limb of facial behaviour research to fully address. Various evolutionary hypotheses thought to explain other individual differences could provide a valuable starting point. Social niche specialisation provides an evolutionary explanation for why individuals do not behave flexibly in the most effective way to suit a given context, but rather show consistent differences in social behaviour to allow them to occupy and exploit a specific niche^74^. Indeed, the interaction we identified between agreeableness and expressivity in predicting reward outcome could hint at the use of facial expressivity as a tool to acquire tangible resources only for those tending towards a more agreeable ‘social role’ (i.e., the tactic an individual uses in response to social challenges^73^). While this hypothesis could be worth exploring further, an assessment of the fitness costs of facial expressivity is first needed. Nettle^87^ argued that the individual differences in the Big Five personality dimensions can be explained by a trade-off between different fitness costs and benefits (e.g., extraversion is associated with increased mating success but increased physical risk and decreased parenting effort^87^. Similar trade-offs should be explored in relation to facial behaviour. Equally, an exploration of potential variables that moderate the relationship between facial behaviour and its benefits is needed. For instance, higher expressivity may only result in positive social outcomes when combined with particular social skills to increase the likelihood that facial behaviour is used appropriately. Finally, further understanding of the developmental trajectory of facial expressivity could provide insight into the causes of population-level variation. These are all promising yet unexplored avenues for future research.

Some of our findings contribute to debates around competing theories of facial expression. Proponents of the dominant Basic Emotions Theory argue that facial expressions are largely automatic outpourings of emotion^88^, which could generate the prediction that more expressive people have poorer facial control. We found mixed support for this, as more facially expressive participants had poorer facial inhibitory control, but were better able to voluntarily produce facial behaviour to effectively achieve social goals in the social tasks. This in some way supports the Behavioural Ecology View of Facial Expression which emphasises the role of facial behaviour in social influence^18,23^. Importantly, we observed a lot of facial behaviour which was not captured by the standard emotional categorisations. The prototypical emotional expressions comprised a low proportion of facial behaviour (consistent with previous research^89,90^) with the exception of smiling, and participants produced these expressions a similar amount across differing social contexts. This seems inconsistent with the primary role of facial behaviour being emotional communication, as their self-reported experience of the social contexts reflected differing valence (e.g., disapproval in the conflict context). Additionally, while a previous meta-analysis found that neuroticism negatively predicted emotional expressivity^91^, we found it to positively predict facial expressivity, suggesting the two are independent constructs. While our findings do not negate emotion influencing some aspects of facial behaviour, they emphasise that the overwhelming focus on emotional configurations in the literature may result in a narrow and biased view of facial expressivity that is not representative of typical behaviour within social interaction.

The ethological approach we have used here overcomes the limitations of subjective measures, and also highlights the complexity in quantifying real-world behaviour. While we captured objective measures of frequency and diversity of facial movements, there are a myriad of other potentially meaningful facial behaviours to explore, such as the speed of onset and offset of facial movements, and the latency of facial responses to social partners. We hope our study stimulates an exploration of other features of facial behaviour using similar approaches. We also observed variations in the clustering of the facial expressivity measures we used between the two studies. In Study 1, a single component was identified, while in Study 2, two components emerged. This discrepancy could be attributed to the larger sample size in Study 2, enabling more precise partitioning of variance. Notably, both components exhibited relatively high communality values, suggesting that these measures constitute a unified component with two subcomponents. However, we recommend future research with large samples to corroborate this observation. We also note that the more controlled nature of our first study yielded higher effect sizes than the more uncontrolled approach in the second study, which is unsurprising given the plethora of factors that may contribute to real-life phenomena such as inter-personal affiliation (e.g., personality compatibility, physical attractiveness, social status), creating noise in the data. Given that the sample size required to control for all of these factors is likely unrealistic in a single research study, we advocate a combination of semi-controlled and uncontrolled naturalistic approaches.

It is time to move towards a unified theory of facial behaviour. Facial behaviour is an integral component of social interaction, yet despite decades of research, studies such as ours which document individuals using facial communication spontaneously with a social partner are scarce. Only by measuring behaviour embedded in a social interaction can we understand how it manifests in a real-world context. Observation of natural facial behaviour has the potential to provide valuable insight into human social dynamics. We hope that our study serves as an example of balancing a naturalistic yet structured approach, which future studies can use to fully explore the causes and consequences of facial communication among other social phenomena.

### Ethical approval

All methods received ethical approval from Nottingham Trent University Schools of Business, Law and Social Sciences Research Ethics Committee (application number: 2021/312). All methods were performed in accordance with the relevant guidelines and regulations and informed consent has been obtained from all participants. Informed consent was obtained from all those whose image appears in the manuscript and/or their legal guardian(s) for publication of identifying information/images in an online open-access publication.

### Supplementary Information

Supplementary Information.

## Data Availability

The datasets and R scripts generated and analysed during the current study are available in the OSF framework (https://osf.io/74x5z/?view_only=5c52185cba2c4f50b5187689683f9ed3). The raw video files used in Study 2 can also be requested at https://betterup-data-requests.herokuapp.com/.
