# Supplementary material for: Being facially expressive is socially advantageous
Source: Sci Rep. 2024 Jun 13;14:12798. doi: 10.1038/s41598-024-62902-6 (PMC11176176; doi:10.1038/s41598-024-62902-6)
Supplement: Supplementary file 1 — Supplementary Information. [file 41598_2024_62902_MOESM1_ESM.docx]

**Supplementary Information**

Table of Contents

[Supplementary information 1 – Consent and Instructions 1](#_Toc143772831)

[1a) Consent form (video call) 1](#_Toc143772832)

[1b) Video Call Invitation 3](#_Toc143772833)

[1c) Consent form (elicited tasks study) 3](#_Toc143772834)

[1d) Consent form (raters study) 6](#_Toc143772835)

[1e) Zoom call script 7](#_Toc143772836)

[1f) Video clip instructions (elicited social tasks) 13](#_Toc143772837)

[Supplementary information 2 – Manipulation Checks 14](#_Toc143772838)

[Supplementary Information 3 – Initial study aims, and excluded details 15](#_Toc143772839)

[Supplementary Information 4 – Missing Data 20](#_Toc143772840)

[Supplementary Information 5 - PCAs 21](#_Toc143772841)

[Supplementary Information 6 – Emotion 24](#_Toc143772842)

[Supplementary Information 7 – Diversity Score calculation 24](#_Toc143772843)

[Supplementary Information 8 – Additional results 26](#_Toc143772844)

## Supplementary information 1 – Study Summary Table, Consent and Instructions

### 1a) Consent form (video call)

This research is being carried out by researchers at the [masked for double blind review], with the purpose of measuring and understanding how people differ in aspects of communication during social interaction.

It will take approximately 45 mins and you will be paid £5 plus bonuses up to £3 upon completion (TOTAL: £8.00)

Your participation will involve a video and audio-recorded video call between you and another participant via Zoom which will be followed by answering questionnaire measures. **You must use a webcam on a laptop/computer for the video call, and not a phone or tablet.**

Your identity will remain anonymous to the other participant and in all of our records. The recording of the video call and your responses to the questionnaires will be kept for use as data in the study.

If you do not wish to take part at any point, please exit the study by closing the browser. Please note that only participants who complete the video call and the questionnaire will receive payment.

**About the Video Call**

The video call will last around 25 minutes

The call will be video and audio recorded, meaning that everything you say and do during the call will be recorded. The call will involve you socially interacting with another participant. You are encouraged to interact with them naturally as if meeting a new colleague or acquaintance. A researcher will be present at all times and will be available to contact via written chat. The researcher will give some instructions via the written chat, and will guide you through the stages of the call. You may be asked to do some of the following at some stage during the call:

- Chat with your partner

- Rate your partner on a character trait

- Be rated by your partner on a character trait

- Come to an agreement with your partner on how to share a reward

- Keep a still face

- Recreate simple behaviours from images (e.g., smile)

You will be free to withdraw from the call at any time without any reason given, though will only receive payment if you complete the call and questionnaire. You will be free to pause the video call for privacy by turning off your video and microphone, and will still receive payment upon completing the call and questionnaire. The researcher will provide guidance on how to do turn your video and microphone on and off prior to the video call.

The researcher will ensure your screenname is anonymous prior to the call so that your identity is not revealed.  You and the other participant are asked not to reveal your identity or contact details or to ask questions about the other’s identity or contact details. You do not have to share any information you don’t feel comfortable with. Your partner will not have any way to contact you after the video call. Please avoid controversial topics that might make your partner uncomfortable.

Directly after the video call you will receive a link the questionnaire.

**About the Questionnaire**

You will be asked to complete a series of questions about your communication, emotions, self-awareness and social attitudes. There will be three attention checks. The questionnaire portion will take approximately 20 minutes. Following this you will receive a debriefing form with further information about the study.

**About the Data Collected**

Data extracted from the video call and your responses to the questionnaire measures will be collected for use in the study, for publication in a scientific journal. No images or videos of you will be published in a journal or in science communication without your prior consent. Data will be stored securely, and will be anonymised. You can contact the researchers within 2 weeks of participating to withdraw your data from the study without any reason given.

**CONSENT**

Please click the statement below to indicate your consent 
(If you do not consent to any of these statements, you may exit the study by closing your broswer)

- I agree to participate in the study
- I agree to attend the Zoom call after completing this form today using a laptop/computer (and not a tablet or phone)
- I understand that I will be video and audio recorded during the video call, and that the recording will be stored and used for data in the study
- I agree to take part in the questionnaire
- I understand that I can withdraw at any time from the study without reason
- **OPTIONAL PERMISSIONS:**
- Do you consent for your data to be used in future studies unrelated to the present study?
- Do you consent for still images or video clips of you participating in the video call to be used in science communication or in journal publications?

### 1b) Video Call Invitation

- The video call must take place **on a laptop or computer** from the Zoom app.
- Go to a spot with good lighting and stable internet
  - *Light source should be in front of you, not behind you.
- Remove anything that may obscure the face (e.g., wear contact lenses instead of glasses).
- Ensure your face is fully visible throughout (you may be asked to move position to improve visibility).

Attending the Zoom Call:

- Please immediately use the following link to attend the call on your laptop/computer:
  - <link provided here>
  - Click ‘open zoom.us’ and launch the call from the Zoom application.

### 1c) Consent form (elicited tasks study)

*You will need a working webcam for this study*

 We thank you for agreeing to participate for a second time in our study. This research is being carried out by researchers at [masked for double blind review], with the purpose of understanding facial expression and developing a questionnaire to measure facial expressivity. The last time you participated, you completed a questionnaire asking questions related to your facial behaviour, and took part in a Zoom video call.

*What this study involves*

In this study, we will ask you to re-take some of this questionnaire, and to answer further questions about your facial behaviour and social environment. There is no need to remember how you answered before, or to try to mimic, or diverge from, your previous answers. Please just respond to the questions as best you can as they apply to you now. There will be some attention checks. You will also be asked to record short video clips which record your facial behaviour. You need a functioning webcam to take the videos. These video clips, along with clips from the original Zoom call, will be rated by third party observers on various qualities (e.g., how much you appear to be listening to another person). You will receive bonus payments if you upload the video clips correctly and according to the instructions. Your responses will be anonymised.

*About the data stored and your rights and freedoms*

All University research is reviewed to ensure that participants are treated appropriately and their rights respected. This project has been considered by [masked for double blind review] Ethics Committee and has met with a favourable ethics opinion. Further information can be found at: [masked for double blind review]

You will be free to withdraw from the study at any time without any reason given, though will only receive payment if you complete the full study. Data from your responses to the questionnaire measures and video clips will be collected for use in the study, for publication in a scientific journal.

The University undertakes research as part of its function for the community under its legal status. Data protection allows us to use personal data for research with appropriate safeguards in place under the legal basis of public tasks that are in the public interest. The only personal data we obtain from you is your personal image in the video recordings; we do not have access to other identifying information such as your name. Data will be stored on [masked for double blind review] secure servers and only members of the research team will have access to the video recordings during the project using their [masked for double blind review] login details. The data may be stored locally on password-protected laptops available only to researchers for processing, but video recordings will be deleted from the laptops once processing is complete.

[MASKED FOR DOUBLE BLIND REVIEW] will be responsible for all of the data during the study. Once the study is over, data that would allow others to check and verify our findings will be transferred to [MASKED FOR DOUBLE BLIND REVIEW]’s data archive, where it will be kept for at least ten years. Any anonymous data, which could not lead to the identification of either you or your organisation, will be publicly available. This will allow anyone else (including researchers, businesses, governments, charities, and the general public) to use the anonymised data for any purpose that they wish, providing they credit the University and research team as the original creators. Below you will be given the option to consent to your data being used for future research unrelated to the current study. If you agree to this, your video recordings will be available to the researchers after the current study is over. If you choose not to consent to this, your video recordings will be destroyed after the current study is complete. You will also be given the option to agree to video or still images from your video clips to be published in science journals or used in science communication. If you consent, these clips may then be made publicly available, which is irreversible, although you can at any time request for future use of these images to be halted for these purposes. You can contact the data protection officer within 2 weeks of participating to withdraw your personal information without any reason given, but the research data may be retained as part of the study.

The researcher's details are as follows: [masked for double blind review]

Please indicate your consent below to continue with the study

- I agree to take part in the questionnaire and to record video clips of facial behaviour for use as data in the study.
- I consent to these uploaded video clips, as well as clips from the original Zoom call, to be rated by third party observers.
- I understand that I can withdraw at any time from the study without reason.
- I agree for my data to be used in future studies unrelated to the current study (optional)
- I agree for video or still images from my uploaded video clips to be published in scientific journals or in science communication (optional)

### 1d) Consent form (raters study)

Thank you for agreeing to participate.

In this study, you will be viewing short video clips and will then be asked to respond to some questions about the video clips you see.

You will then also be asked to complete some short questionnaires related to your social circle and personality.

It will take approximately 45 minutes and you will receive payment of £4.88 for your time.

There will be attention checks to ensure you are paying attention, and failure to respond correctly to these may result in loss of payment.

**Consent**

This research is being carried out by researchers at [masked for double blind review], with the purpose of understanding facial expression and developing a questionnaire to measure facial expressivity.

**What this study involves**

You will be asked to rate video clips of people according to a number of criteria (e.g., to indicate the degree to which the person appears to be listening). You will also be asked to complete some short questionnaires related to your social circle and personality. This will take approximately 45 minutes and you will receive £4.88 for your time. There will be some attention checks, and failing to pass these may result in losing your payment. Your responses will be anonymised.

**About the data stored and your rights and freedoms**

All University research is reviewed to ensure that participants are treated appropriately and their rights respected. This project has been considered by [masked for double blind review] Research Ethics Committee and has met with a favourable ethics opinion. Further information can be found at: [masked for double blind review]. You will be free to withdraw from the study at any time without any reason given, though will only receive payment if you complete the full study. Data from your responses will be collected for use in the study, for publication in a scientific journal. The University undertakes research as part of its function for the community under its legal status.  We do not collect any personal information of yours. Data will be stored on [MASKED FOR DOUBLE BLIND REVIEW] secure servers and only members of the research team will have access to the data. [MASKED FOR DOUBLE BLIND REVIEW] will be responsible for all of the data during the study. Once the study is over, data that would allow others to check and verify our findings will be transferred to [MASKED FOR DOUBLE BLIND REVIEW]’s data archive, where it will be kept for at least ten years. Any anonymous data, which could not lead to the identification of either you or your organisation, will be publicly available. This will allow anyone else (including researchers, businesses, governments, charities, and the general public) to use the anonymised data for any purpose that they wish, providing they credit the University and research team as the original creators. Below you will be given the option to consent to your data being used for future research unrelated to the current study. If you agree to this, your anonymised data will be available to the researchers after the current study is over. If you choose not to consent to this, your data will be destroyed after the current study is complete. You can contact the data protection officer within 2 weeks of participating to withdraw your data without any reason given.

The researcher's details are as follows: [masked for double blind review]

The Data Protection officer's details are as follows: [masked for double blind review]

Please indicate your consent below to continue with the study:

- I agree to take part in the study
- I understand that my responses in the study will be used as data for publication in scientific journals.
- I understand that I can withdraw at any time from the study without reason.
- I consent for the data I provide to be used in future studies unrelated to the present study (optional)

### 1e) Zoom call script

At the beginning of the call, the participant did not have visual or audible access to the confederate. In the written chat, participants received instructions from the researcher which participants could respond to via written chat when required. The instructions first directed the participant to practise turning on and off their microphone and camera, to ensure they could do so efficiently when asked at various points throughout the call, and to ensure their face was clearly visible in the video image. The participant was then connected visually and audibly to the confederate during a video and audio-recorded interaction. Below is the script used by the researcher as as a guide through the video call. Red text indicate instructions to self. Black italics indicates text sent to the participant via the written chat. Black bold text indicates words spoken aloud to participant.

BEFORE CALL

Make sure powerpoint presentation is visible correctly, with splitcam as the selected camera

Researcher laptop volume is on

Participant named ‘anon1’, and researcher named ‘researcher’

Participant video is off

Timer ready.

Make sure my chat is big enough.

OPENING INSTRUCTIONS

*Change screenname to ‘ANON2’.*

**Click ‘chat’ on the bottom of the screen – The written chat should be on the right of the screen**

*Wait for response.*

*Is this written chat on the right of your screen?*

*Wait for response.*

*Welcome to the study!*

*Please keep an eye on this written chat to ensure that you don’t miss instructions.*

*Respond to all questions I ask via this written chat.*

*Your screenname has been changed temporarily to ‘ANON2’ for your privacy*

*Wait for response.*

*What is your Prolific ID code?*

*Wait for response.*

*Are you on a laptop or computer?*

*Wait for response.*

*Are you accessing the call from the Zoom app?*

*Wait for response.*

*Now let’s practise turning your video and microphone on and off.*

IF VIDEO OFF:

*Now turn on your video; click the ‘Start video’ button on the bottom left of the screen.*

*(Directions to stay in view, good lighting).*

*Thank you – try to stay in this position in full view for the duration of the call.*

IF VIDEO ON:

*Now turn off your video; click the ‘Stop video’ button on the bottom left of the screen.*

IF AUDIO OFF:

*Now turn on your audio; press the ‘Join Audio’ (or ‘Unmute’) button on the bottom left of the screen. Then please speak the word ‘testing’ out loud.*

IF AUDIO ON:

*Now turn off your audio; press the ‘mute’ on the bottom left of the screen*

*TURN OFF SOUND*

(guide through any difficulty).

*Are you confident you can turn the video and audio on and off when asked?*

*Wait for response.*

*The call will be recorded, but if you ever want privacy, you can turn your video and audio off.*

*Wait for response.*

*During the call make sure your face is clearly visible at all times (e.g., don’t touch your face with your hands, drink from a cup etc.)
The call is about to begin. For the first 3 minutes, just get to know your partner. For example, you could talk about your experiences with using Prolific. Then I will give more instructions.
Please wait until I add your partner to the call.*

Add person in

Click ‘chat’

Press record.

NEUTRAL CONDITION

*You are now being recorded. Please ensure your video and microphone are turned on.*

*For 3 minutes; get to know each other (for example, you could talk about your experiences with using Prolific)*

Set timer for 2 minutes

Turn on participant audio and video

Line up next text in chat

Wait 2 minutes

Consistent uninterrupted speaking (listening condition)

*Both participants: please turn off your video and mute your microphone now (click the ‘mute’ button and the ‘stop video’ button on the bottom left).*

CONFLICT CONDITION

<Private chat>

*Next, you will be deciding together how a bonus monetary reward will be divided between the two of you.*

*Are you happy to discuss this with your partner and decide this together?*

*Wait for response.*

*Please turn your video back on and unmute your microphone (by pressing the ‘unmute’ and ‘start video’ buttons on the bottom left of the screen).*

<Public chat>

*You must now decide how to divide the bonus you have been given.*

*Out of 100% of the reward, what percentage should each of you get?*

*Please discuss now, and type your answer into the written chat when you have decided.*

*LINE UP NEXT TEXT*

*You have decided that ANON1 will get % of the reward****.*** *This means you BOTH receive* *% of the reward. ANON1 was asked to convince ANON2 to get a higher proportion of the reward, because this would determine how much you both would get.*

*LINE UP NEXT TEXT.*

*(Discuss)*

*Now please can you both turn your videos off and mute your microphones (by pressing the ‘mute’ and ‘stop video’ buttons on the bottom left of the screen).*

*Wait for response.*

AFFILIATION CONDITION:

*Now you can earn another bonus reward. For the next 3 minutes, you and your partner will be chatting again*, *but one of you will rate the other on a character trait at the end.*

<Private chat>

*You have been assigned the role of ‘RATEE’. Your partner will rate you on a character trait at the end of the next 3 minutes. Your partner will NOT be told what the character trait is yet, but I will tell you now.

The character trait is LIKEABILITY. You will get more money if your partner rates you high in LIKEABILITY.*

*You should now chat for 3 minutes, and try to get your partner to rate you high in LIKEABILITY to earn a bigger bonus.*

*Do NOT discuss anything that I have just told you with your partner. Do you understand?*

*Wait for response.*

(wait and clarify any misunderstandings).

*Please turn your video back on and unmute your microphone (by pressing the ‘unmute’ and ‘start video’ buttons on the bottom left of the screen).*
*Please continue to chat for the next 3 minutes.*

Set Timer

Wait 2 minutes

*Mute microphone, apologise*

*Now; ANON1 must tell a joke.*

*Tell joke.*
*Please can you both turn your videos off and mute your microphones (by pressing the ‘mute’ and ‘stop video’ buttons on the bottom left of the screen).*

*Wait for response.*

*Inhibition Condition*

**Rate how well you got on from 0-10.**

*<Private chat>*

*How well did you get on with your partner? Please give a score from 0-10. This will not affect your payment in any way.*

*Wait for response.*

*There is no bonus reward for the next task. First please ensure you are in ‘speaker view’. Go to the top right of the screen and click ‘View’. Then select ‘Speaker view’. Have you done this?*

*Get 1-minute timer ready*

*For this task, you should try NOT to move your face or head. Your partner will try to make you react, while you try to keep a straight face/head. It is VERY IMPORTANT that you keep watching your partner’s face. You shouldn’t use a strategy to keep a still face that involves ignoring or looking away from your partner. Do you understand?*

*Wait for response.*

*The task will begin immediately after your video turns on – keep a straight face and do not talk for one entire minute, no matter what your partner does or says.*

*Wait for response.*

*Please turn your video back on and unmute your microphone (by pressing the ‘unmute’ and ‘start video’ buttons on the bottom left of the screen).*

Start timer

***Ok, first I’m just going to watch you; I’m sure you’ll move your face. (Hold up timer: at 15 seconds continue). Now I have something to tell you while you try to keep your face straight. I am not a participant in this study. I have been posing as a participant but I am in fact a researcher in this study. PAUSE. The truth is, I intentionally turned my microphone off while continuing to speak to see how you would react, and I intentionally told a joke that was not funny to see if you would still laugh. PAUSE. The monetary bonuses you receive are not dependent on any performance in the tasks; you will be given the maximum possible bonus, and so will receive the maximum payment upon completion of the entire study.***

***1 minute is up – the task is now over and you can relax your face.***

PRODUCTION CONDITION

***I now have to inform you that I am not a participant in this study. I have been posing as a participant but I am in fact a researcher in this study. The reason we chose to not reveal this to you in advance was because we wanted to record as natural a reflection of peer social interactions as possible, and felt that knowing that I was a researcher might change your behaviour or put pressure on you to perform a certain way. The truth is that you were not being rated on likeability; we told you this as we wanted you to feel motivated to affiliate. The monetary bonuses you received were not dependent on any performance in the task; you will be given the maximum possible bonus, and so will receive the maximum payment upon completion of the entire study. So at this point I want to check in with you to see if me posing as a participant has caused you any distress?***

**(discuss).**

***There is one more portion of the video call left, then I will give you a link to the questionnaire.***

***For the last section, I will show you a powerpoint presentation, and ask you to make some facial movements. I’m going to go off camera now*** *(but don’t mute)*
*(show presentation now)*

***First, click ‘view’ on the top right hand corner, and select ‘gallery mode’. All three panels should be equal sizes. Is that ok?***

*Wait for response.*

***Please follow the instructions on the presentation: try to make ONLY the movements in the images shown as much as possible.***

(Go through presentation, giving instructions where necessary)

<Give link to questionnaire>

SAVE CHAT

### 1f) Video clip instructions (elicited social tasks)

| Table S1 – Video clip instructions in the elicited social tasks | | | |
| --- | --- | --- | --- |
| **Actors’ social goal** | **Type** | **Instructions for actors** | **Video example** |
| Look friendly | Greeting | Please record a video clip of you acting out a greeting. Speak into the camera and say the words "Hello, how are you?" as though you are greeting another person (no other words). Try to making a friendly impression (you will be rated on this) | **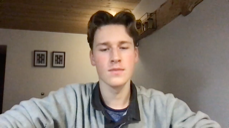** |
| Appear to be listening | Listening | Please record a video clip of pretending to listen attentively to a friend for 10 seconds. Try to show that you are listening (you will be rated on this)  Please ensure that the recording is at least 10 seconds. | **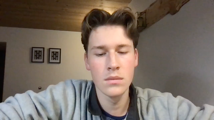** |
| Try to reassure | Reassure | Please record a video clip of you pretending to reassure another person. Speak into the camera and say: "Don't be embarrassed, it's ok"  *Try to reassure the other person by giving a kind expression* (you will be rated on this) | **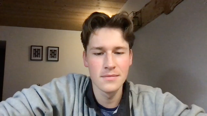** |
| Look threatening | Disagree (Threat) | Please record a video clip of a disagreement. Say "No, I do not agree with that" (no other words). *Try to communicate disapproval, in a way that threatens the other person.* (you will be rated on this) | **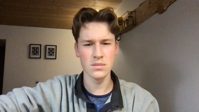** |
| Be liked despite disagreement | Disagree (Like) | Please record a video clip of another disagreement. Say "No, I do not agree with that" (no other words)  *Try to communicate disapproval, but without making the other person dislike you.* (you will be rated on this) | **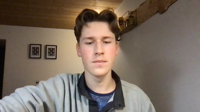** |

## Supplementary information 2 – Manipulation Checks

No participant strongly agreed with the statement *“I knew my partner was not another participant before she informed me”,* with just 14.29% indicated some agreement (mean: 1.80, SD: 1.05, min: 1, max: 4). Note that we conducted all analyses using a reduced sample of participants who did not show agreement with this statement and results were not substantially different; all significant models maintained significance using this reduced sample. However, more conscientious participants were more facially expressive in this reduced sample (r(39)=.33, *p=*.04). Given that this did not replicate in the full sample or in Study 2 data we did not explore this finding further. Similarly, no participant strongly disagreed with the statement *“Conversation with my partner felt natural; similar to meeting face-to-face”* with just one participant indicating some disagreement (0.2%)*,* and 94.23% indicating at least some agreement *(*mean: 4.32, SD: 0.64, min: 2, max: 5)*.* Additionally, participants appeared to be very familiar with the use of video calls, with 98.08% using video calls at least once a month. Together these indicate that the video call elicited a naturalistic interaction between the participant and the confederate. The majority of participants (71.32%) indicated at least some agreement with the statement *“I disapproved of my partner suggesting the reward be divided unevenly”* (mean: 3.84, SD: 1.13, min: 1, max: 5), indicating the uneven reward suggestion elicited a conflict of interest (note that in addition, only those who disagreed with the initial offer of 20% of the reward were included in any conflict analyses). The vast majority (88.46%) indicated at least some agreement with the statement *“I was motivated to make a good impression after being told my partner would rate me on likeability”* (mean: 4.48, SD: .79, min: 2, max: 5) indicating that participants were affiliative in the affiliation condition. However they also appeared to be affiliative in the neutral condition, as 84.62% indicated at least some agreement with the statement *“I was motivated to make a good impression in the opening conversation”* (mean: 4.31, SD: 0.89, min: 1, max: 5)*.*

## Supplementary Information 3 – Initial study aims, and excluded details

Nationalities of video call participants in Study 1 included:

- Czech Republic: 1
- France: 1
- Germany: 2
- Greece: 2
- Hungary: 1
- India: 2
- Ireland: 2
- Israel: 1
- Italy: 3
- Latvia: 1
- Netherlands: 1
- Nigeria: 1
- Poland: 5
- Portugal: 6
- South Africa: 2
- Spain: 1
- Sweden: 1
- Turkey: 2
- United Kingdom: 15
- United States: 2
- Venezuela, Bolivarian Republic of: 1

Nationalities of rater participants in Study 2 included:

- Angola: 1
- Argentina: 1
- Austria: 1
- Canada: 4
- Chile: 5
- Czech Republic: 3
- Ecuador: 1
- France: 1
- Germany: 4
- Greece: 9
- Hungary: 10
- India: 2
- Ireland: 2
- Italy: 12
- Latvia: 2
- Mexico: 5
- Netherlands: 1
- Norway: 1
- Pakistan: 1
- Peru: 1
- Poland: 30
- Portugal: 26
- Russian Federation: 1
- Rwanda: 1
- Slovenia: 1
- South Africa: 8
- Spain: 5
- Turkey: 1
- United Kingdom: 22
- United States: 6
- Venezuela, Bolivarian Republic of: 1
- Vietnam: 1
- Zimbabwe: 1

The video call experiment was initially designed to validate a self-report questionnaire measuring individuals’ facial expressivity. The aim of the video call was to create a naturalistic social interaction, during which the participants’ facial behaviour would be quantified and compared with responses to the newly developed questionnaire. The three main conditions (neutral, affiliation, conflict) were included to capture a range of behavioural contexts, and aligned with the contextual elements of the items in the questionnaire. The shorter conditions were included as they related directly to particular items of the questionnaire (e.g., the aim of the listening condition was to measure the participant’s facial behaviour while listening, in order to compare it to questionnaire items such as “*People can tell I am listening to them from my facial expression”*).

The aims of the current study were designed after all video call data had been collected (but prior to the follow up and rating components), so the confederate had no motivation to adjust her like score or concede the reward negotiation according to the participant’s facial behaviour. The confederate’s like score was measured along with the participants’ rating of the confederate to test whether rapport between participants could explain difficulty to inhibit facial behaviour in the inhibition condition (it did not). The reward outcome was not of interest.

*Elements of procedure not relevant to current study*

Part 1 - video call and questionnaire

Following the video call, as indicated in the main text, participants (actors) were asked to indicate their agreement to statements related to the video call. The full list of statements (in order of appearance) were as follows:

- I knew my partner was not another participant before she informed me.
- I was surprised when I found out my partner was not another participant
- I was motivated to make a good impression in the opening conversation
- I was motivated to make a good impression after being told my partner would rate me on likeability
- Conversation with my partner felt natural; similar to meeting face-to-face
- I was motivated to be polite after being told my partner would rate me on likeability
- When my partner was speaking at length, I was listening
- I found my partner's joke funny ("How do you make a piece of paper dance? You put a little dance in it")
- When my partner's microphone was muted but she kept speaking: I was annoyed
- When my partner seemed embarrassed that her microphone was muted: I tried to reassure her
- When my partner seemed embarrassed that her microphone was muted: I felt sorry for her
- I disapproved of my partner suggesting the reward be divided unevenly
- I found the discussion about splitting the reward difficult
- I found it difficult to keep a straight face when my partner was just watching me
- I found it difficult to keep a straight face when she was telling me she was not another participant.

They were then also asked how often they use video calls in general (e.g., with work colleagues, family or friends, using any video call software), from a scale of 1-5 (*Never* to *Very often – more than once per week*).

They were then asked to complete a newly-designed 41-item facial expressivity questionnaire, with 5 attention checks, prior to completing the 10-item personality questionnaire. They then also completed the BEQ, followed by the self-awareness questionnaire and social desirability questionnaire before being debriefed.

Part 2 - Follow-up

Participants were shown the same gifs of facial movements they had viewed in the initial video call, but asked to self-report their ability to make the facial movement. They were then given the same facial expressivity questionnaire as in the initial study, but with 37 new questions added. They were then asked to upload video clips as outlined in the main texts. The first video clip participants were asked to upload was of them speaking about their hobbies. They were asked: *Please record a video clip of you speaking about your hobbies for 30 seconds. Speak naturally as though you were meeting a new friend. Please ensure that the recording is at least 30 seconds.*They were then asked to upload the other 6 video clips outlined in the main text. Following this they completed the social network index (SNI) and were asked to respond to eight questions about their satisfaction with their facial behaviour and social network before being debriefed.

Part 3 – Rating

Raters responded to additional questions upon viewing video clips, which were used to satisfy the aims of the initial study, but are not relevant for the current study. After viewing clips from the conflict condition of the video call, raters were additionally asked “*Does this person look friendly?”,* and “*Is this person managing the conflict well?”*After viewing clips from the embarrassment condition of the video call, raters were additionally asked “Do you think this person has reassured their partner?” and “Is this person giving a kind expression?”

After viewing the uploaded greeting video clips, the raters were additionally asked “*As a first impression, how much do you like this person?”* After viewing the uploaded disagree (like) clips, the raters were additionally asked “*Does this person look threatening?*” and after viewing the uploaded disagree (threat) clips, the raters were additionally asked “*As a first impression, do you like this person?*”

Raters also rated the hobbies video, and were asked:

“*As a first impression, how much do you like this person?” and* *“How well do you feel you can read this person (i.e., have an idea of what is on their mind, what their intentions/motivations are)?"*

After rating all video clips, raters also completed the SNI, and the 10-item personality inventory.

## Supplementary Information 4 – Missing Data

All FACS data was excluded from 1 participant as iMotions could not reliably detect the face, although they provided sufficient clips to be included in readability and competence measures. In addition, 1 participant was excluded from the inhibition condition as imotions couldn’t detect the face. 3 participants were excluded from the embarrassment condition as iMotions couldn’t detect the face. Conflict data were excluded from 3 participants, as no conflict occurred (they agreed with getting a lower amount with no protest). 6 participants were excluded from the controlled AU repertoire measure (including the three missing from all conflict) as they didn’t meet the criteria of 25 AUs in each of the three contexts.

19 actors were excluded from the competence scores as they did participate in the follow up study. 5 actors were excluded from readability and perceived readability scores as they did not give permission for use of their data in future studies and so their videos could not be shown to the raters.

6 raters failed 30% or more of the attention checks in the rating study and were excluded from the sample (although these are already excluded from the sample size reported in the text).

## Supplementary Information 5 - PCAs

*Study 1*

We performed a total of five PCAs on the six AU measures; four from study 1 and one from study 2. In the first PCA the measures were calculated across contexts as outlined in table 3 in the main text. In the subsequent three PCAs, calculation of measures was restricted to each of the three main contexts; neutral, affiliation and conflict. In the PCA from study 2, measures were calculated across the whole five minute clip. Measures correlated considerably (see figures below).

We chose PCAs as some Kaiser-Meyer-Olkin (KMO) test values were low, and the resulting structures from PCAs and principle axis factor analysis are almost identical (Horn, 1965). Bartlett’s tests of sphericity indicated it was appropriate to conduct PCAs. Inspection of scree plots and use of parallel analyses (Horn, 1965) indicated extraction of a single component should be retained in all four analyses from study 1, while they indicated extraction of two components in the study 1 analysis.

| **Study 1** | |
| --- | --- |
| 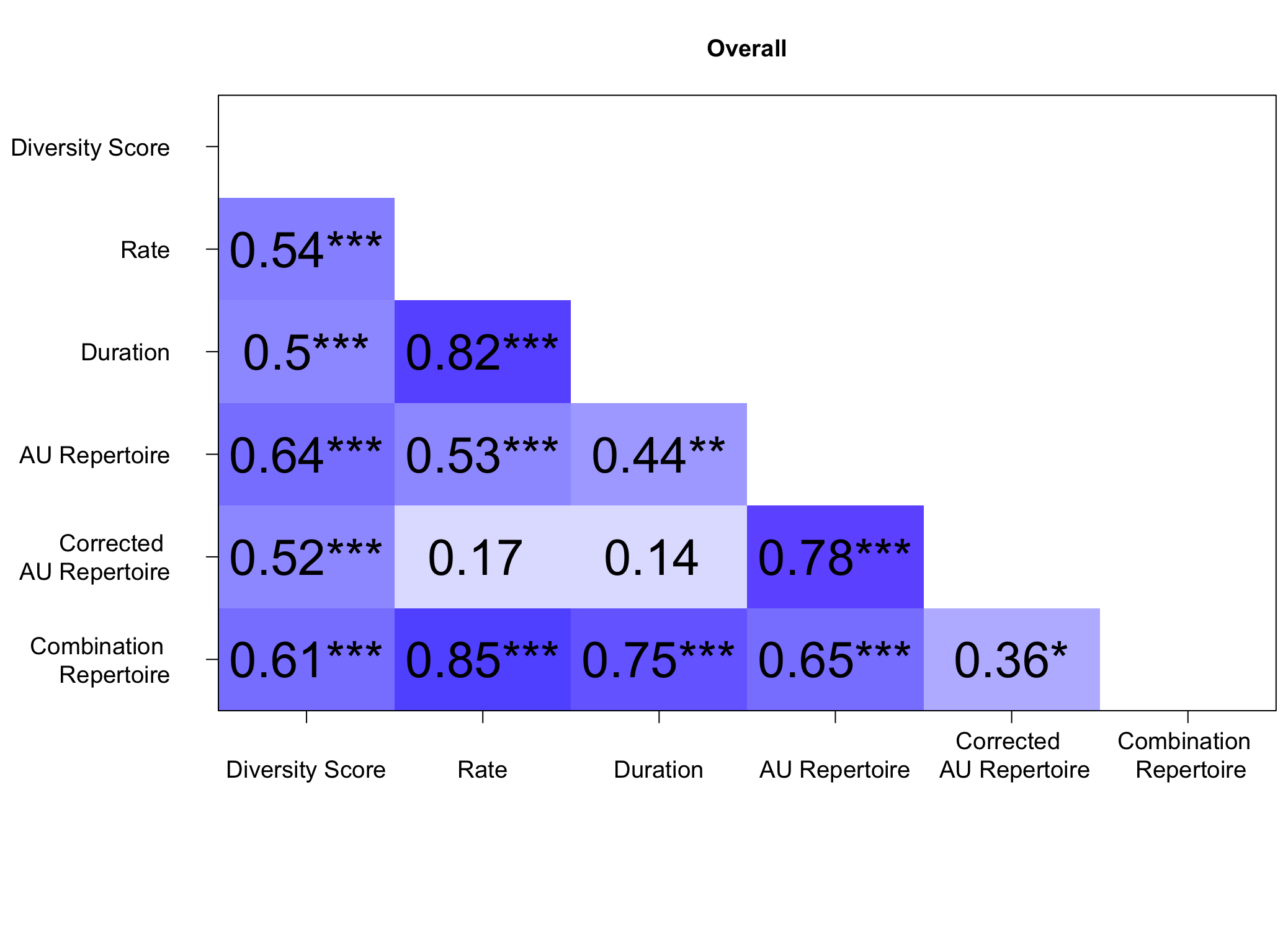 | 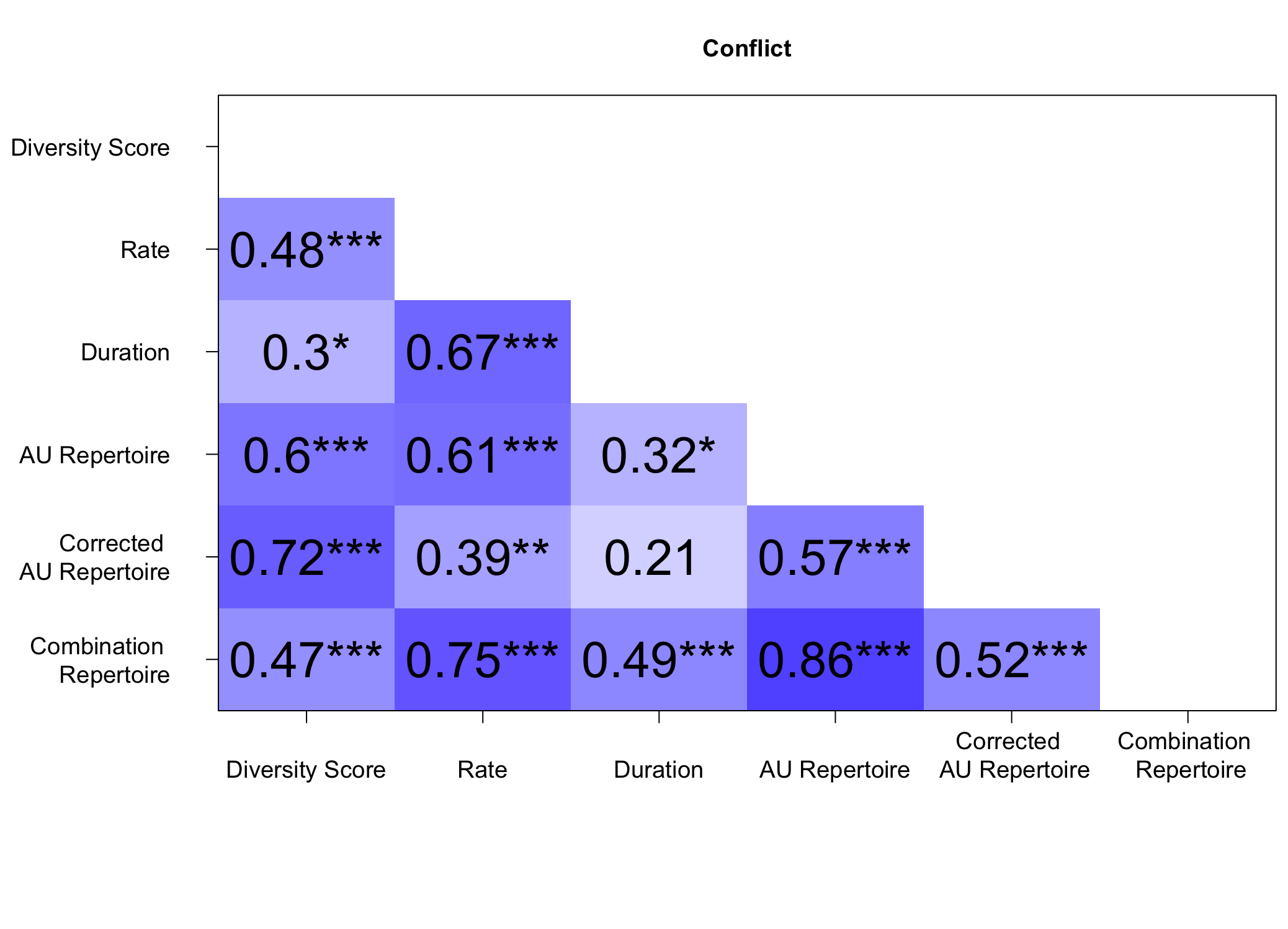 |
| 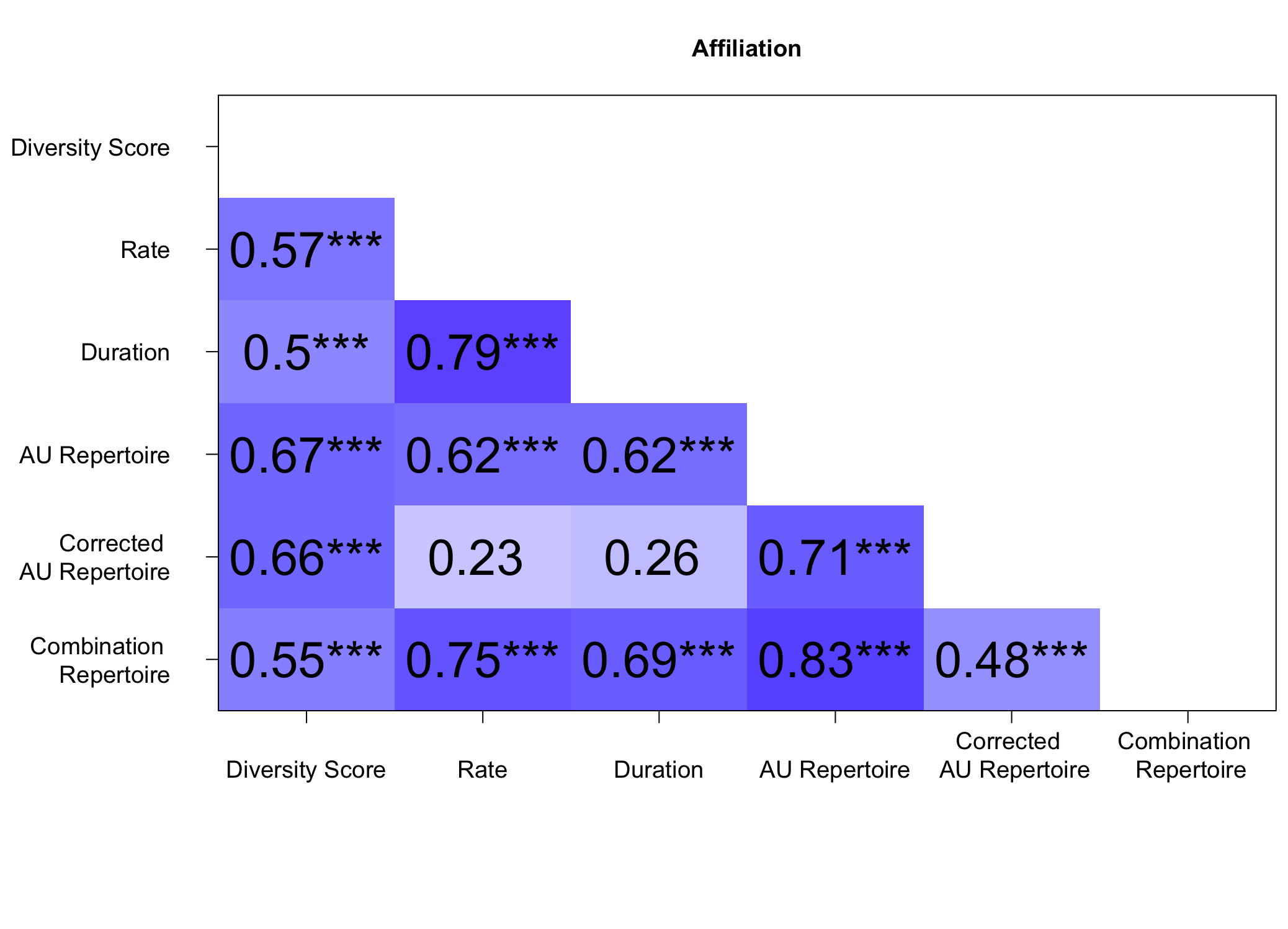 | 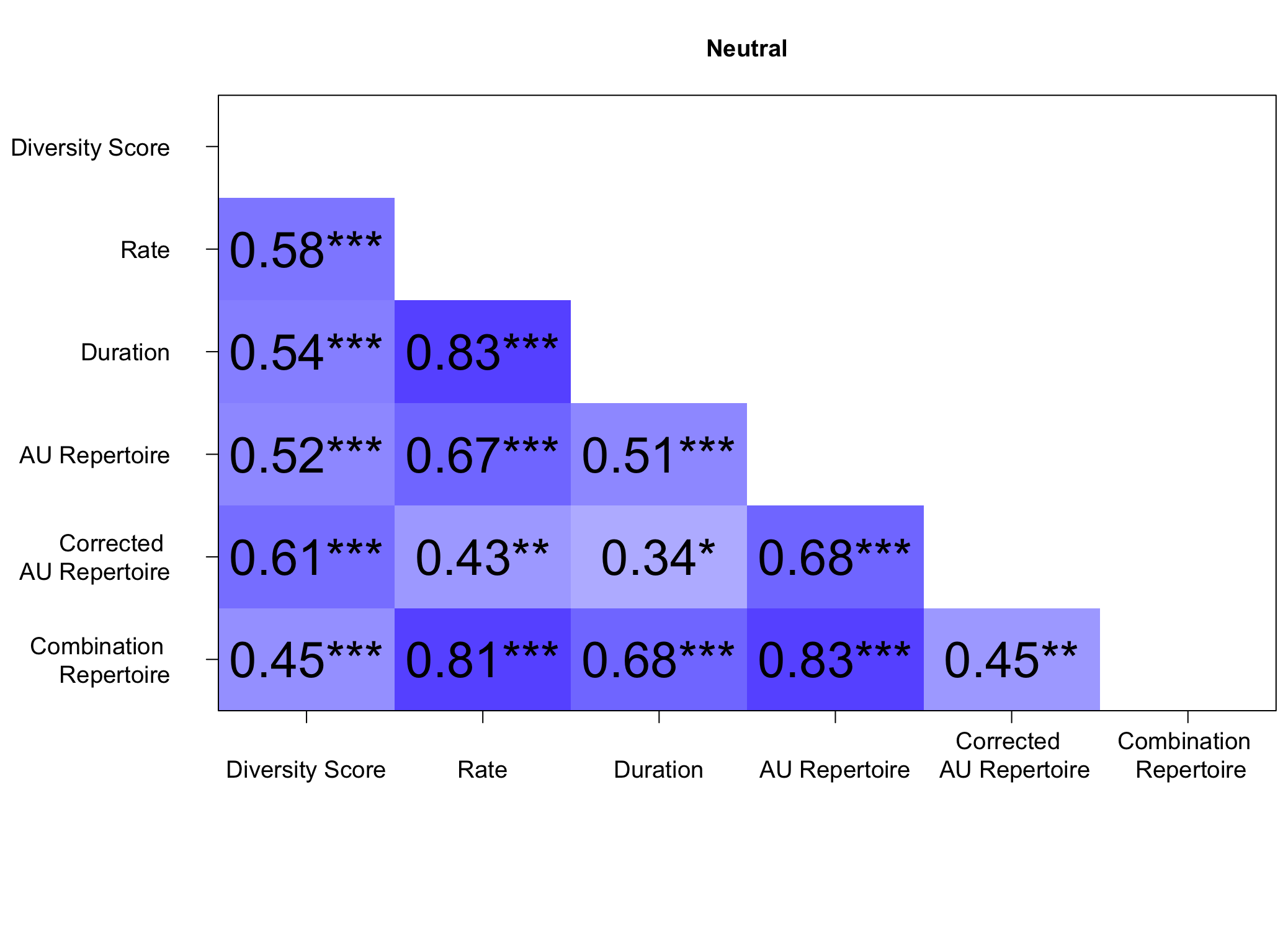 |
| **Study 2** | |
| 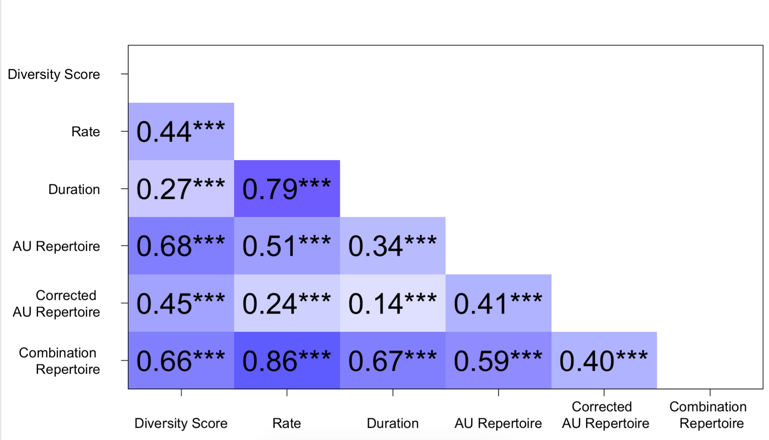 | |
| *Figure S1 –* Correlations between FACS measures. Values indicate Pearson’s R.  ** = p*<.05  *** = p*<.01  *** = *p*<.001 | |

| Table S2 – Results of five PCAs | | | | | |  |  |
| --- | --- | --- | --- | --- | --- | --- | --- |
|  |  | Study 1 (all 1 component) | | | | Study 2 |  |
|  |  | Overall | Neutral | Affiliation | Conflict | Component 1 | Component 2 |
|  | **Variance explained by component** | 64% | 67% | 67% | 62% | 42% | 37% |
| **Variable loadings on component** | Diversity Score | .80 | .74 | .80 | .75 | .29 | .83 |
|  | Rate | .85 | .90 | .82 | .83 | .92 | .26 |
|  | Duration | .79 | .80 | .80 | .61 | .92 | .04 |
|  | AU Repertoire | .83 | .87 | .91 | .86 | .35 | .75 |
|  | Corrected AU Repertoire | .58 | .70 | .66 | .72 | .00 | .79 |
|  | Combination Repertoire | .90 | .88 | .89 | .89 | .78 | .51 |
| **Bartlett’s test of sphericity** | χ^2^ (5) | 918.20 | 990.72 | 945.84 | 891.01 | 28100 | |
|  | *p* | <.001 | <.001 | <.001 | <.001 | <.001 | |

## Supplementary Information 6 – Emotion

https://blog.affectiva.com/emotion-ai-101-all-about-emotion-detection-and-affectivas-emotion-metrics


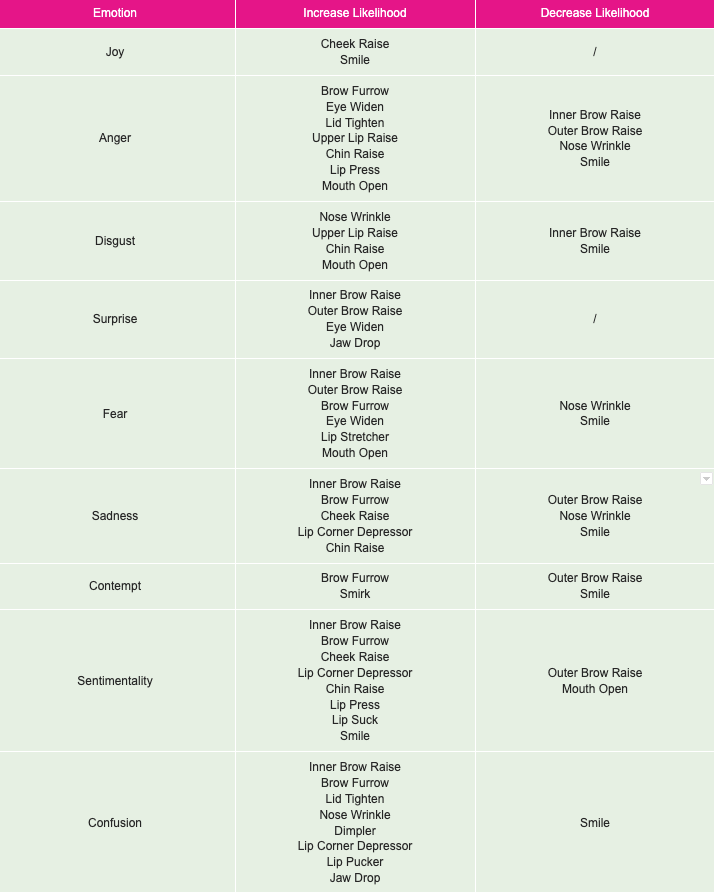


## Supplementary Information 7 – Diversity Score calculation

From Scheider et al., (2013 pp. 4):

Diversity score… “takes into account both the repertoire and the rates. It should be interpreted as a weighted repertoire. The diversity measurement incorporates information about how many types of facial expressions are observed and how evenly those types are represented [Hill, 1973]. For a given number of types, the value of a diversity index is maximized when all types are equally present. In other words, the more different types there are and the more they are evenly represented, the higher the diversity measurement. Thus, if the number of facial expressions of an individual is given by S, we first calculated the Shannon Information [Shannon, 1948] for the n‐ individual as:


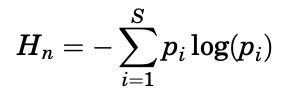


Here pi represents the ratio between the number of each facial expressions and the total number of facial expressions for a given individual. The diversity of facial expressions is given by:


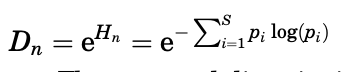


The corrected diversity index (Dnt) [Hill, 1973] of the facial expressions for each individual is then calculated by:


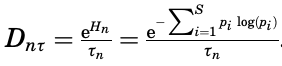


*References*

Hill MO. 1973. Diversity and evenness: a unifying notation and its consequences. Ecology 54:427–432.

Scheider, L., Liebal, K., Oña, L., Burrows, A., & Waller, B. (2014). A comparison of facial expression properties in five hylobatid species. *American Journal of Primatology*, *76*(7), 618-628.

Shannon CE. 1948. A mathematical theory of communication. Bell System Technical Journal 27:379–423.

## Supplementary Information 8 – Additional results

Note that for each video clip, the raters scored perceived readability directly after liking, so we considered that the high correlation between perceived readability and raters’ liking score may be falsely inflated due to ease of selecting similar scores close together. However, we found that raters did not consistently score ratings close together similarly; for instance there was no relationship between raters’ liking of actors in the conflict video clip and their ratings of the actors’ disapproval of the reward (r(44)=-.087, *p*=.564). We also calculated new liking scores using just one half of the raters’ responses, and new perceived readability scores using the other half of the raters’ responses, and the correlation between these independent scores was still high (r(45)=.844, *p<.*001). These suggest that the strong positive relationship between perceived readability and raters’ liking score is likely to be valid.

| **Table S3**  **Repeated Measure ANOVAS; comparing expressivity measures across contexts.** | | | | | | | | | |
| --- | --- | --- | --- | --- | --- | --- | --- | --- | --- |
|  |  | **Full sample** | | | **Excluding influential datapoints** | | | | |
| **Measure** | **Correction applied** | **df** | **F** | ***p*** | **Correction applied** | **df** | **F** | ***p*** | **N influential datapoints removed** |
| Diversity Score | GGe | 1.34,63.02 | .67 | .058 | NA | 2,93 | 2.397 | .097 | 4 |
| Rate | HFe | 1.83,85.91 | .914 | .025* | HFe | 1.74 | 78.13 | .061 | 4 |
| Duration | HFe | 1.71,80.46 | .856 | .889 | NA | NA | NA | NA | NA |
| AU rep | NA | 2, 97 | .941 | .394 | NA | 2,91 | 0.967 | .384 | 2 |
| Corrected repertoire | NA | 2, 88 | 1.067 | .348 | NA | 2,86 | 0.851 | .43 | 2 |
| Combination repertoire | NA | 2,87 | 1.219 | .300 | NA | 2,82 | 0.908 | .407 | 9 |
| *Note*: where Mauchley’s test (Mauchley’s ) indicated a violation of the assumption of sphericity, corrections were applied to produce a valid F ratio; Greenhouse-Geiser correction (GGe; ) was applied where ε < 0.75 and Huyn-Feldt correction (HFe; ) was applied where ε > 0.75 (Field, 2009; Girden, 1992). Where influential datapoints were present we report results with and without them.  **p*<.05 | | | | | | | | | |

| **Table S4**  **Results from repeated measures ANOVAs, comparing comparing the use of facial expressions of emotion across neutral, affiliation and conflict condition.** | | | | |
| --- | --- | --- | --- | --- |
|  | **Correction applied** | **df** | **F** | **p** |
| *Joy* | GGe | *(1.4,65.73)* | *.699* | *.380* |
| *Surprise* | *NA* | *(2,94)* | *1.006* | *.369* |
| *Anger* | *NA* | *(2,94)* | *1.015* | *.366* |
| *Contempt* | *GGe* | *(1.55,73.07)* | *.777* | *.239* |
| *Disgust* | *GGe* | *(1.51,70.77)* | *.753* | *.274* |
| *Fear* | *GGe* | *(1.38,64.92)* | *.691* | *.616* |
| *Sadness* | *HFe* | *(1.68,79.09)* | *.841* | *.133* |
| *Note*: where Mauchley’s test (Mauchley’s ) indicated a violation of the assumption of sphericity, corrections were applied to produce a valid F ratio; Greenhouse-Geiser correction (GGe; ) was applied where ε < 0.75 and Huyn-Feldt correction (HFe; ) was applied where ε > 0.75 (Field, 2009; Girden, 1992).  **p*<.05 ***p*<.01 | | | | |

|  |
| --- |
| 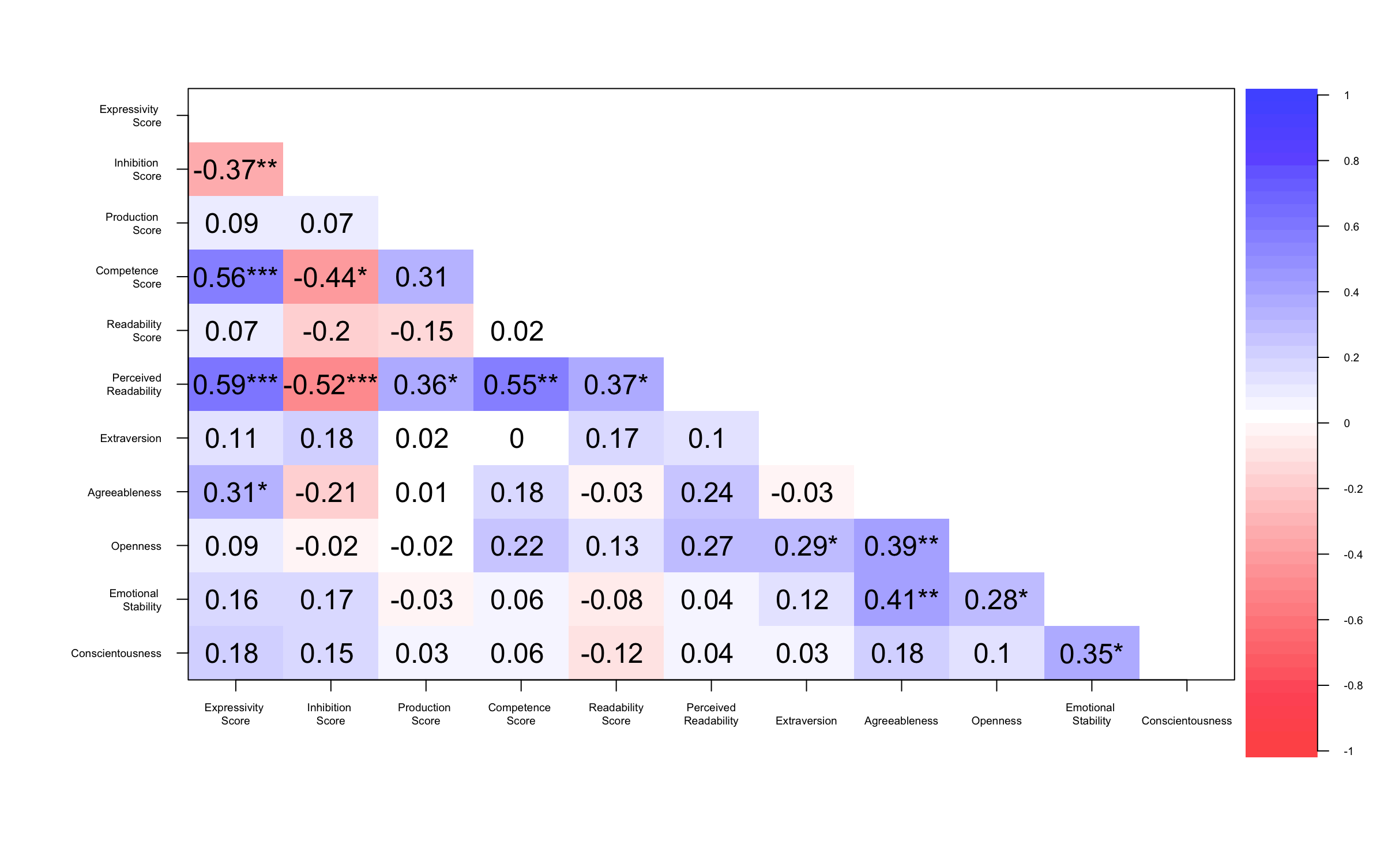 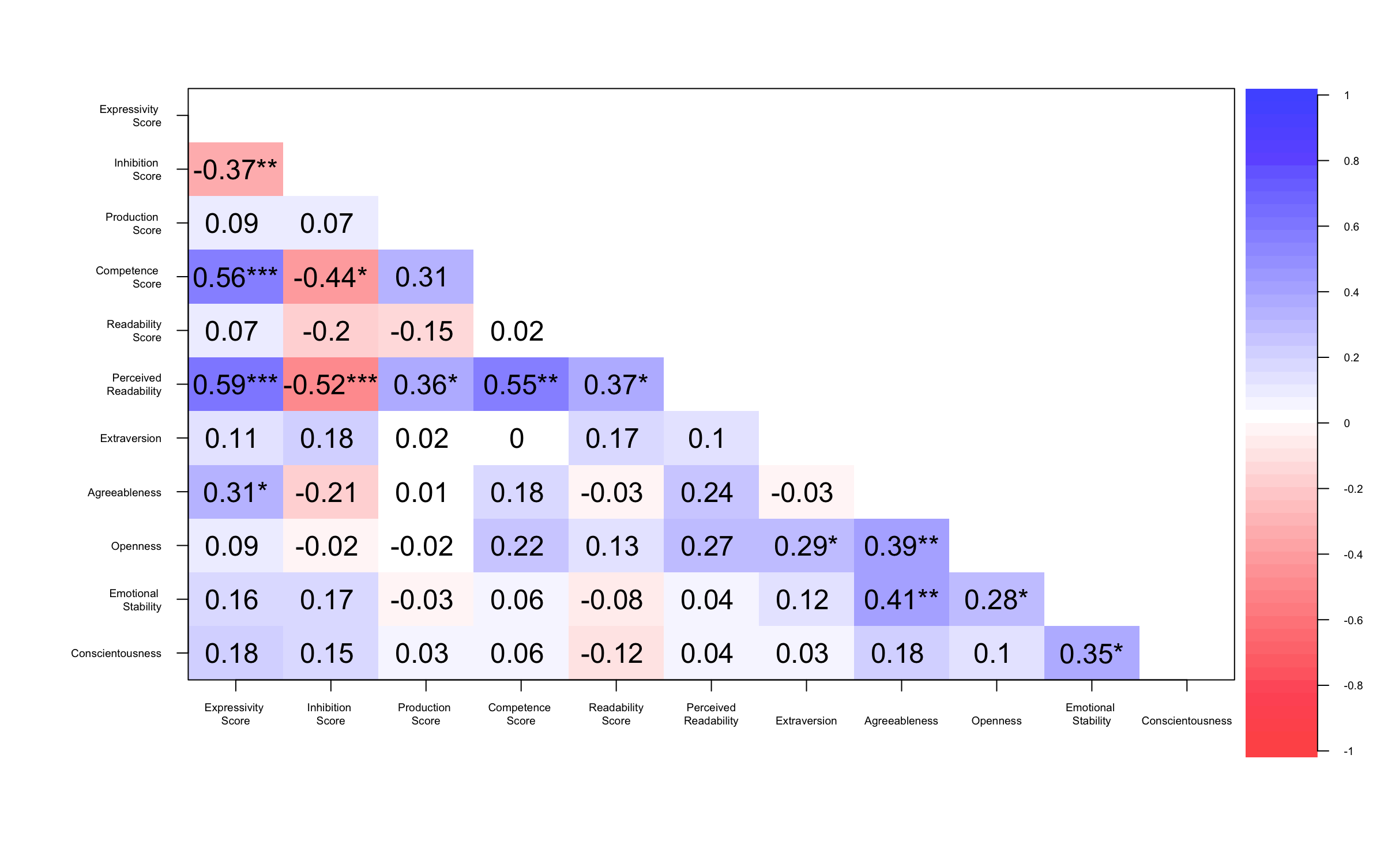 |
| Figure S2 – correlations between facial and personality measures. Numerical values indicate Pearson’s R  ** = p<.05* |

|  |
| --- |
| 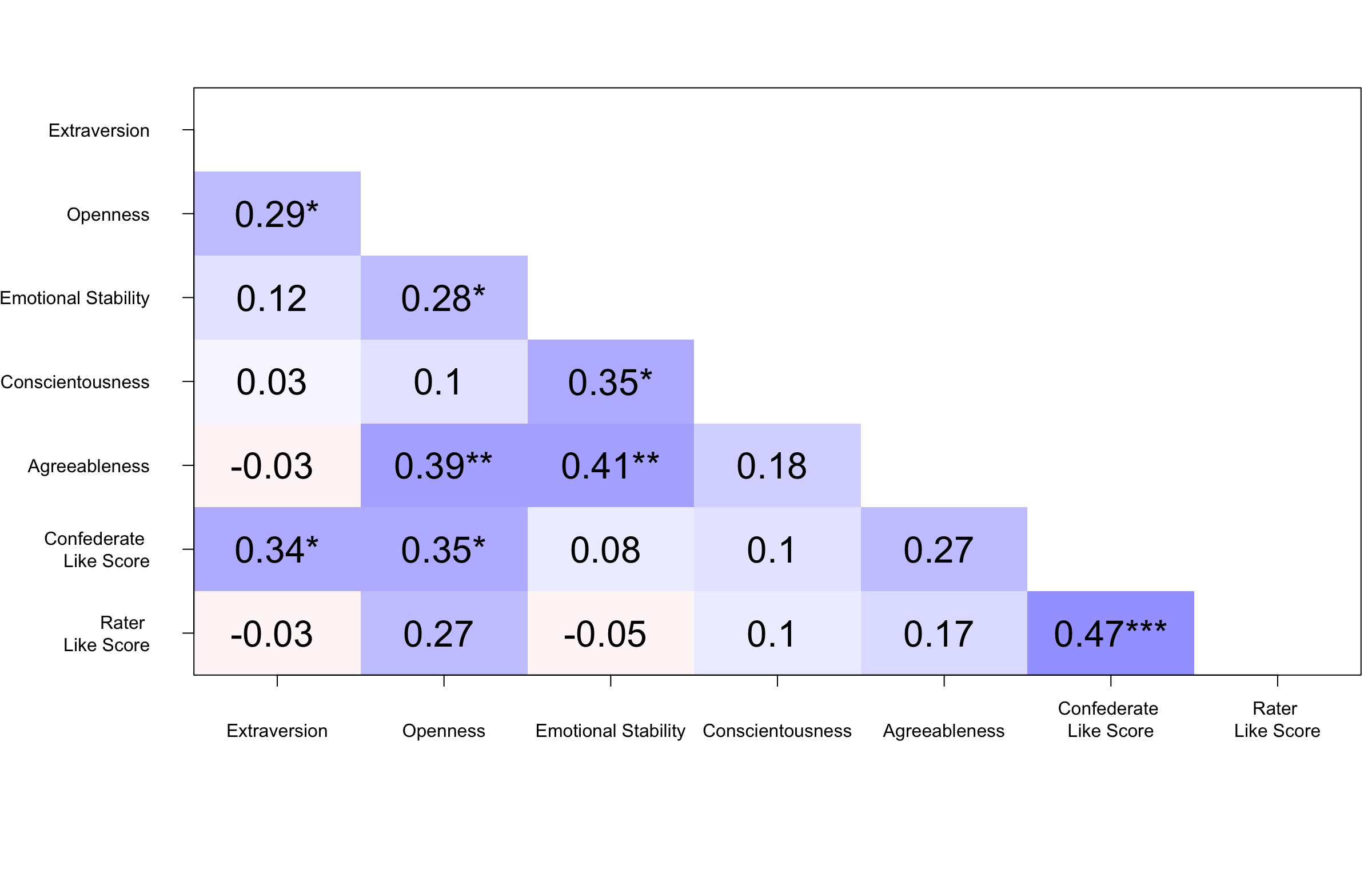 |
| Figure S3 – correlations between like scores and personality measures. Numerical values indicate Pearson’s R |
|  |

*
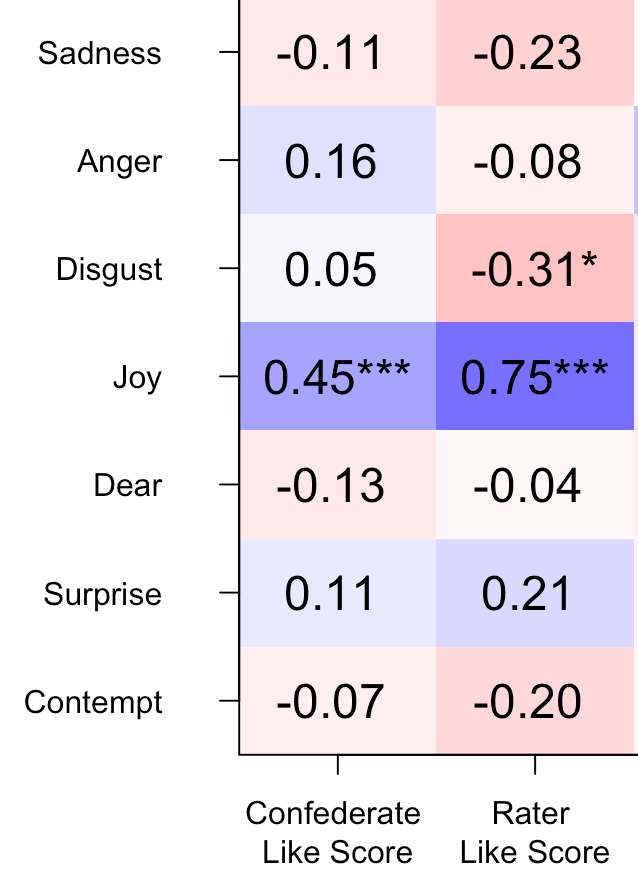
*

Figure S4 – correlations between like scores and emotional expressions. Numerical values indicate Pearson’s R.
